# *Clerodendrum trichotomum* Thunb.: a review on phytochemical composition and pharmacological activities

**DOI:** 10.3389/fphar.2024.1505851

**Published:** 2025-01-06

**Authors:** Linzhen Li, Zhiyi Tang, Shengjia Xiao, Xiangjie Dai, Yutong Wang, Xi Wei

**Affiliations:** ^1^ School of Pharmacy, Guizhou Medical University, Guiyang, China; ^2^ State Key Laboratory of Functions and Applications of Medicinal Plants, Guizhou Medical University, Guiyang, China; ^3^ Research Center for the Development and Application of Ethnic Medicine and TCM (Ministry of Education), Guizhou Medical University, Guiyang, China; ^4^ School Hospital, Guizhou Medical University, Guiyang, China

**Keywords:** *Clerodendrum trichotomum* Thunb., phytochemicals, pharmacological activities, diterpenes, phenylpropanoid glycosides

## Abstract

*Clerodendrum trichotomum* Thunb. (*C*. *trichotomum*) is a shrub or tree of the genus *Clerodendrum*, family Lamiaceae, which is widely distributed in China, Korea, India, Japan and Philippines. *C*. *trichotomum* is a kind of medicinal and edible plant which integrates ecological afforestation, garden greening, herbal medicine and flavor wild vegetable. As a traditional Chinese medicine, *C*. *trichotomum* is used to treat various diseases and conditions, which has inspired research on the pharmacological activities of its different parts, including roots, stems, leaves, flowers and fruits. These studies have revealed many biological properties of *C*. *trichotomum*, such as antihypertensive, antitumor, antioxidant, antiinflammatory, antibacterial, analgesic, sedative, anti-HIV-1 and whitening. A total of 164 secondary metabolites were isolated from *C*. *trichotomum*, and their structural types were mainly terpenoids, flavonoids, steroids, phenylpropanoids and phenylpropanoid glycosides, phenylethanosides, phenolic glycosides, anthraquinones, polyketones, cyclohexylethanoids, alkaloids and acid amides. The presence of a variety of phytochemicals, especially abietane diterpenes, clerodane diterpenes, phenylpropanoid glycosides and flavonoid glycosides, plays an important role in the activity diversity of this plant. The current study is attempt to comprehensively compile information regarding the phytochemicals and pharmacological activities of *C*. *trichotomum*, provide the chemotaxonomic proof for the taxonomic classification of the plant, and also highlight the current gaps and propose possible bridges for the development of *C*. *trichotomum* as a therapeutic against important diseases.

## 1 Introduction

**FIGURE 1:**
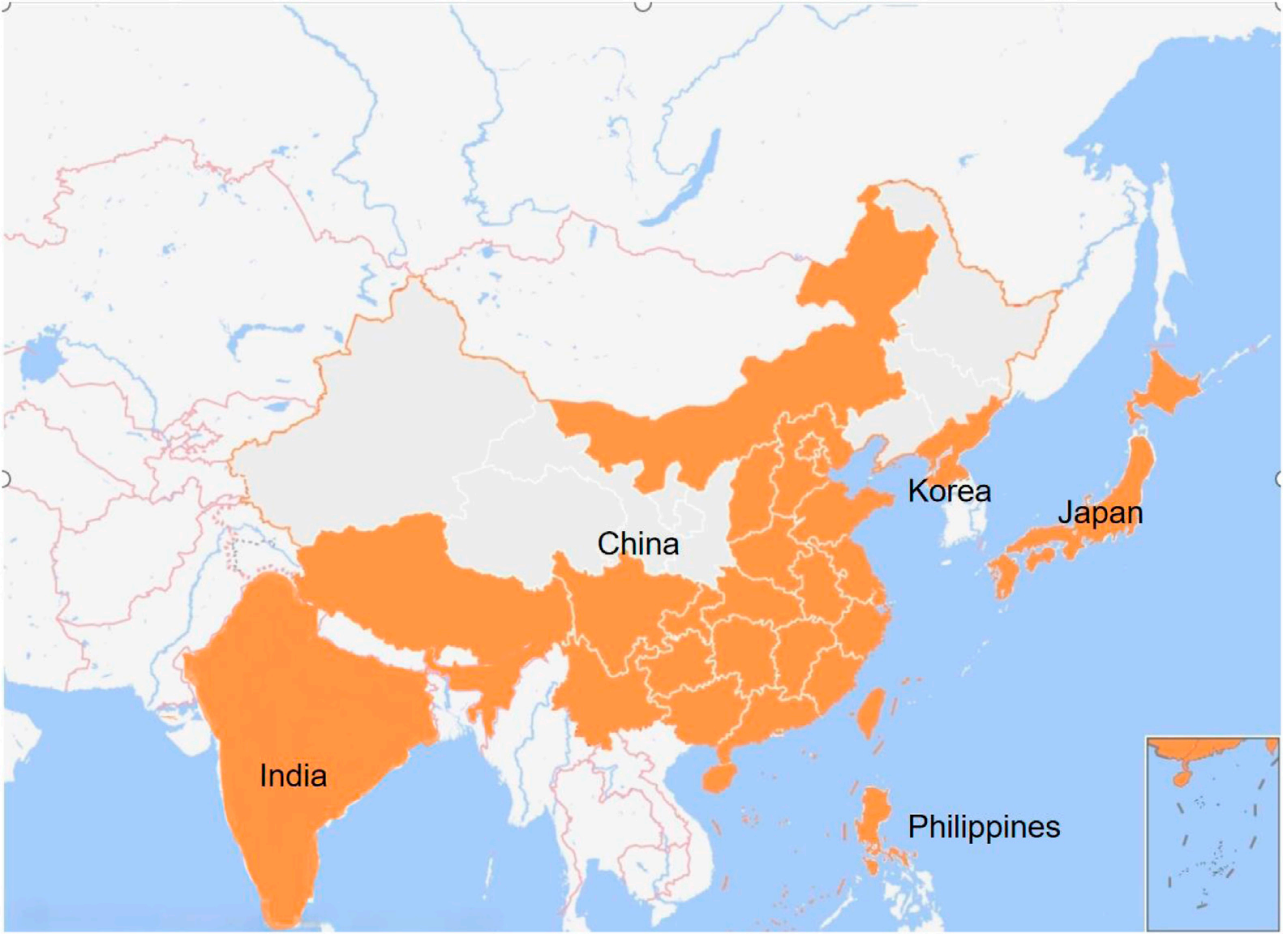
The orange spots in the map depicted the main region of *Clerodendrum trichotomum* Thunb.

*Clerodendrum trichotomum* Thunb. is a deciduous shrub or small tree widely distributed in China, Korea, India, Japan and the northern Philippines (Figure 1), and can be mostly found in hillside scrub below 2,400 m above sea level (Chen and Michael, 1994). The first mentioning of *C. trichotomum* was recorded in the book of *Bencao Tujing* of Song Dynasty. Historically, due to its origin in Haizhou area of Lianyungang, Jiangsu province, it was once used as Changshan (another kind of Chinese medicine *Dichroa febrifuga* Lour.), so it is named Haizhou Changshan in China. It is also known as “Chou Wu Tong,” “Di Wu Tong,” “Ai Tong Zi” and so on (Chinese Materia Medica editorial board, 1999). The flowering period of *C. trichotomum* is from June to October, and the fruiting period is from August to November (Peng and Chen, 1982). The inflorescence is large, the flowers and fruits are gorgeous. Especially, the white flowers, the red calyx and the blue fruit can coexist on the same tree at the same time, and the three colors complement each other. The flowers and fruits can reach a half-year long ornamental period, so it is one of the rare summer flower plants and owes high ornamental value. *C. trichotomum* has strong resistance and large absorption to sulfur dioxide (SO_2_) (Zheng et al., 2006), strong tolerance and enrichment ability to heavy metal arsenic (Mahmud et al., 2008). In addition, it has strong stress resistance to drought, salt, waterlogging, barren, high temperature and so on (Xie et al., 2008; Wei et al., 2009; Xie et al., 2012; Zeng et al., 2013). Therefore, it is a suitable tree for urban landscaping, saline-alkali land greening and abandoned land vegetation restoration.

*C. trichotomum* is mainly used as a folk remedy for the treatment of rheumatism, hemiplegia, hypertension, migraine, malaria and dysentery (Xie, 1996; Chinese Materia Medica editorial board, 1999). Its roots, stems, leaves, flowers and fruits can be used as medicine (Table 1). What’s more, the young leaves of *C. trichotomum* are a kind of green woody wild vegetable favoured by local residents in Guizhou, Hubei, Sichuan, Yunnan and other places of China because of their unique flavor, fresh taste and slightly sweet aftertaste. The leaves are non-toxic, bitter and cold in taste, with special flavor and rich in pectin, vegetable protein and a variety of amino acids. They are essential raw materials for Enshi Tujia to make “fairy tofu,” and have been favored by the majority of consumers because of their unique flavor and natural pollution-free (Wang, 2002), and can also be made into special drinks (Zheng et al., 2013).

**TABLE 1:** Ethnobotany of *Clerodendrum trichotomum* Thunb.

Part use	Disease cure	Country/origin /Area	Reference
Roots	Rheumatism, asthma and other inflammatory diseases (arthritis)	Guang dong	Shrivastava and Patel (2007); Au et al. (2008)
Stems	Inflammatory skin conditions, headaches, hypertension, rheumatism, rheumatic articular pain and rheumatism fever	China, Taiwan, Korea, Japan	Nagao et al. (2001); Kang et al. (2003); Zhang et al. (2022); Ogunlakin et al. (2023); Yang et al. (2024)
Leaves	Asthma, inflammatory skin conditions, headache, hypertension, anti-rheumatic, rheumatism and rheumatic articular pain, rheumatism fever	China, Taiwan, Korea, Japan, Nepal	Nagao et al. (2001); Shrivastava and Patel (2007); Subba et al. (2016); Ogunlakin et al. (2023); Yang et al. (2024)
Flowers	Inflammatory conditions, headache, and hypertension	China, Taiwan, Korea, Japan	Ogunlakin et al. (2023)
Fruits	Anticancer	China, Japan	Nagao et al. (2001); Gomulski and Grzegorczyk-Karolak (2024)
Raw material	Eczema	China	Gomulski and Grzegorczyk-Karolak (2024)
Decoctions	Rheumatoid arthritis, joint pain, numbness, and paralysis	China	Gomulski and Grzegorczyk-Karolak (2024)
All	Anti-diabetic, neuralgia, arthritis, cough, abdominal lump, anti-hypertensive and sedative	China, Korea	Bae et al. (2012); Gomulski and Grzegorczyk-Karolak (2024); Yang et al. (2024)

*C. trichotomum* is a kind of medicine homologous food which integrates ecological afforestation, garden greening, traditional Chinese medicine and flavor wild vegetable. Despite the existence of many bioactive phytochemicals and some potential biological properties, only one recent review of *C. trichotomum* has been published and only 50 references have been reviewed (Gomulski and Grzegorczyk-Karolak, 2024). A complete and comprehensive compilation of the phytochemicals and pharmacological activities of *C. trichotomum* is still absent in the literature. This may be an important reason for the underutilization of this medicinal and edible plant. Hence, in the current study, the comprehensive compilation of phytochemicals as well as pharmacological activities of *C. trichotomum* has been attempted, which also highlights the current gaps and proposes possible bridges for the development of *C. trichotomum* as a therapeutic against important diseases. *C. trichotomum* belongs to the genus *Clerodendrum*. However, it is still debated whether the genus *Clerodendrum* belongs to the Labiaceae family or the Verbenaceae family. Genus *Clerodendrum* was assigned to family Verbenaceae traditionally by Engler and Prantl (Briquet, 1895). This classification method is also adopted in Flora of China (Peng and Chen, 1982; Chen and Michael, 1994). But modern taxonomic systems mostly put it in the family Lamiaceae (Latta, 2008). Therefore, a review of its chemical constituents is of great significance in chemotaxonomy for the scientific definition of its placement in the plant classification system.

## 2 Methodology

**FIGURE 2:**
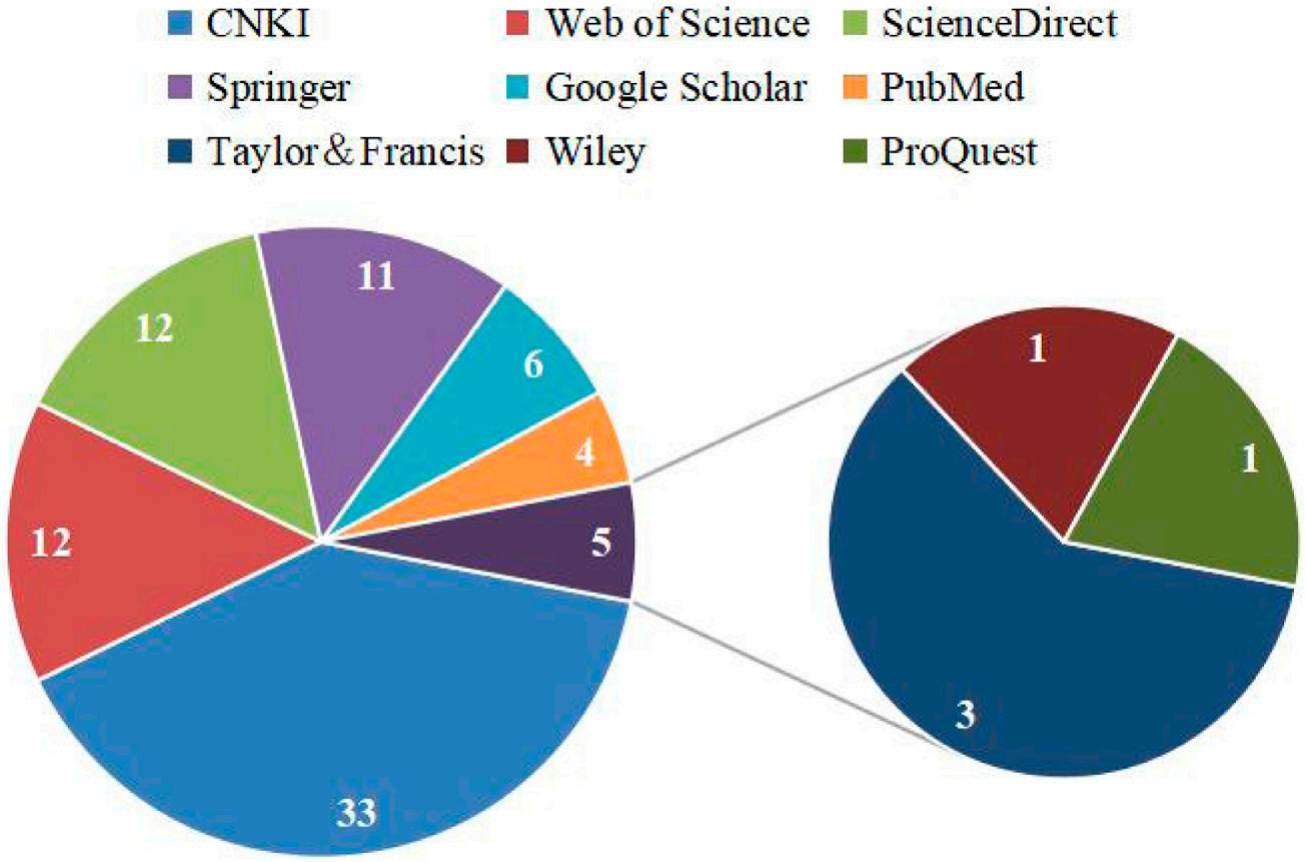
Statistical map of literature database sources.

Information for this review was collected through several popular search engines and databases such as Web of Science, ScienceDirect, Springer, Google Scholar, PubMed, Taylor and Francis, Wiley, ProQuest, CNKI, and classic texts of Chinese herbal medicines (e.g., *Bencao Tujing*), and other websites, such as the Flora of China, the Plant List, YaoZh website (https://db.yaozh.com/). The selection criteria of this article were: 1) Research involves the botany, toxicity and adverse reactions, and the traditional application of *C. trichotomum*; 2) research involves the preparation of crude extract and the separation and identification of monomer compounds; 3) research involves the pharmacological activity of the crude extracts and isolated compounds; 4) research involves the mechanism of action and so on. Keywords used in the literature search were “*C. trichotomum*,” “海州常山,” “phytochemistry,” “chemical constituents,” “pharmacology,” “biological activity,” “traditional uses,” “medicinal uses,” “toxicology,” “safety,” and other related search terms. We searched all eligible literature up to July 2024, with no time and language restrictions. The sources of the literature database were shown in Figure 2. The chemical structures of these compounds isolated from *C. trichotomum* were drawn using the software ChemDraw 22.

## 3 Botanical description

**FIGURE 3:**
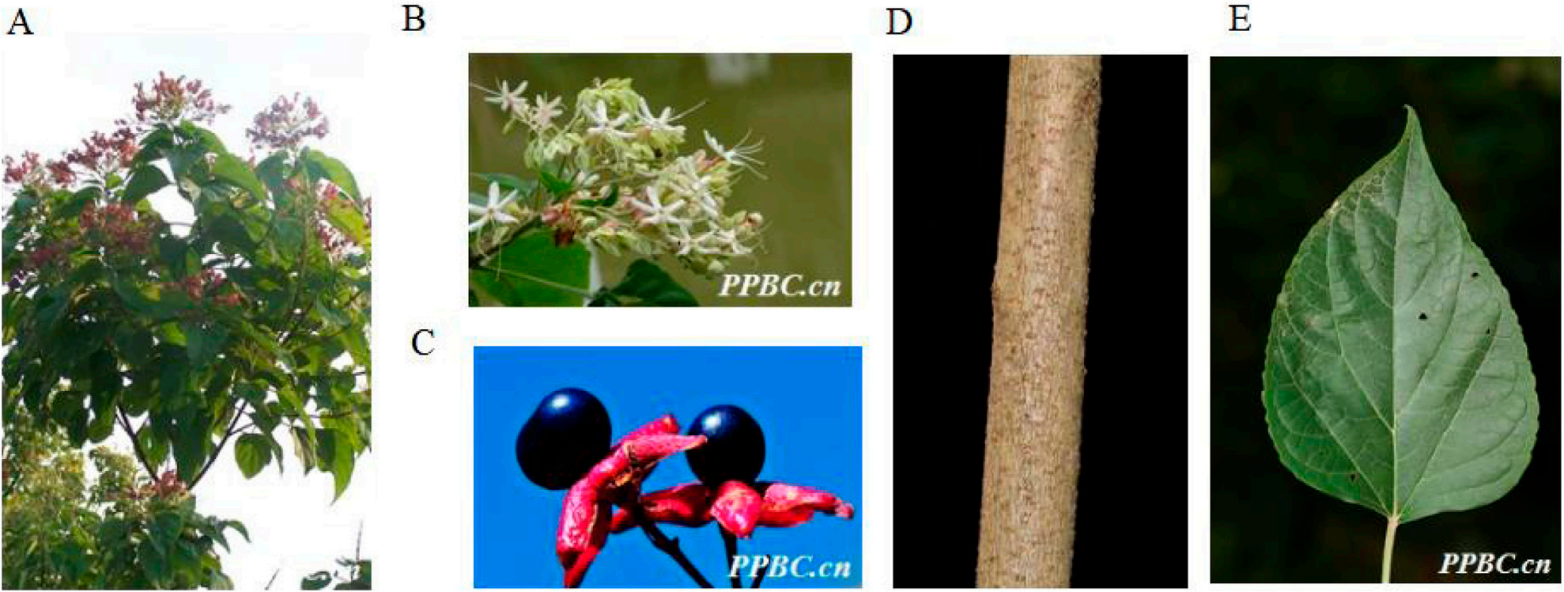
The aerial parts **(A)**, Plant flowers **(B)**, Plant fruits **(C)**, Plant stems **(D)** and Plant leaves **(E)** of *Clerodendrum trichotomum* Thunb (http://ppbc.iplant.cn/).

*C. trichotomum* is a deciduous shrub or tree that belongs to genus *Clerodendrum* family Verbenaceae, 1.5–10 m tall (Figure 3). The branchlets are lenticellate. The leaves of the plant are ovate-elliptic, triangular-ovate or ovate, with a size of around 5–16 cm × 2–13 cm. Their base is broadly cuneate, truncate, or rarely cordate, margin is entire or rarely undulate, apex is acuminate. The leaf blade is greenish abaxially and dark green adaxially. The veins are 3–5 pairs and the petiole is 2–8 cm long. The inflorescences are axillary or terminal, lax, corymbose cymes, dichotomous. The length of inflorescence is 8 and 18 cm and the peduncle is 3–6 cm long. The bracts are elliptic and the flowers are fragrant. The calyx is greenish firstly, then becoming purple, deeply 5-lobed; the lobes are triangular-lanceolate to ovate. The corolla is white or pinkish, with about 2 cm long, its tube is slender; its lobes are oblong, with the size of around 5–10 mm × 3–5 mm. The style is shorter than stamens, both exserted. The drupes are blue-purple, subglobose, ca. 6–8 mm in diam (Peng and Chen, 1982; Chen and Michael, 1994).

## 4 Toxicity and adverse reactions

Limited toxicity research on *C. trichotomum* has been conducted in the literature, although *C. trichotomum* leaves and stems have been used as a medicine in different populations. The median lethal dose (LD_50_) of *C. trichotomum* water extract in mice was 20.6 g/kg by intraperitoneal injection (Yan et al., 1957). Rats were given 150 g/kg hot water (80°C) maceration extract by intragastric administration, and no animal death was observed within 72 h; The LD_50_ for intravenous injection was 19.4 g/kg (Xu and Xing, 1962). Mice were intraperitoneally injected with clerodendronin A (an active compound of *C. trichotomum*), the LD_50_ was 1.84 g/kg (equivalent to crude drug 370 g/kg); Intraperitoneally injected mice with clerodendronin B (another active compound of *C. trichotomum*), the LD_50_ was 3.21 g/kg (equivalent to crude drug 550 g/kg) (Xu et al., 1960a). The rats were given 0.25 g/(kg·d) and 2.5 g/(kg·d) hot water (80°C) maceration extract of *C. trichotomum* by intragastric administration for 60 days, separately, no abnormal changes were observed in growth, blood, urine and pathology except for quiet, reduced activity and loose stool in a few animals (Xu and Xing, 1962). When dogs were given 10 g/(kg·d) for 3 consecutive weeks, there were no significant effects on liver function, blood image, electrocardiogram and pathological examination of heart, liver and kidney, but the dose above 20 g/kg could induce vomiting (Wang et al., 1960).

## 5 Phytochemical investigation

The roots, stems, leaves, flowers and fruits of *C. trichotomum* can be used as medicinal parts. 164 phytochemicals have been reported in the roots and aerial parts of *C. trichotomum* (Table 2). Most of these studies were focused on leaves, stems, roots and fruits, while few papers have been done on the flowers so far. The prime objective behind the phytochemical identification in *C. trichotomum* was the discovery of phytochemicals responsible for the reported biological activities. Hence, in several studies, further biological properties were also analyzed after the phytochemical studies. Various important phytochemicals discovered in these studies may explain the reported biological properties of *C. trichotomum*. Among 164 secondary metabolites, there were 74 terpenoids (including 4 monoterpenes, 3 sesquiterpenes, 51 diterpenes and 16 triterpenes), 11 flavonoids, 16 steroids, 24 phenylpropanoids, 3 phenylethanosides, 2 phenolic glycosides, 3 anthraquinones, 2 polyketones, 7 cyclohexylethanoids, 7 alkaloids, 2 acid amides, and 13 other compounds (including 3 acids, 3 alcohols, 3 aldehydes, 2 esters, 1 alkane and 1 peroxide). In addition, the volatile oils from different parts of *C*. *trichotomum* also have been analyzed and reported.

**TABLE 2:** Compounds isolated from *Clerodendrum trichotomum*.

No.	Compound name	Part of plant	Fraction	Type of extract	References
Monoterpenes					
1	Loliolide	Leaves	PE	95%EtOH	Xu et al. (2015)
2	Melittoside	Stems	*n*-BuOH	85%EtOH	Li et al. (2022a)
3	8-Epiloganic acid	Stems	*n*-BuOH	85%EtOH	Li et al. (2022a)
4	Neohancoside A	Flowers	EtOAc	MeOH	Lee et al. (2016)
Sesquiterpenes					
5	Clovane-2*β*,9*α*-diol	Leaves	*n*-BuOH	95%EtOH	Xu et al. (2015)
6	Annuionone D	Leaves	PE	95%EtOH	Xu et al. (2015)
7	Corchoionoside C	Leaves	PE	95%EtOH	Xu et al. (2015)
Diterpenes					
8	Ferruginol	Stems	PE	85%EtOH	Zhang et al. (2022)
9	Cyrtophyllone B	Stems	EtOAc	85%EtOH	Li et al. (2014a)
10	Sugiol	Stems	EtOAc	85%EtOH	Li et al. (2014a)
11	(4*S*,5*S*,10*S*)-12-(*β*-D-glucopyranosyl)oxy-11-hydroxyabieta-8,11,13-triene-19-oic acid *β*-D-glucopyranosyl-(1→6)-*β*-D-glucopyranosyl ester	Stems	*n*-BuOH	85%EtOH	Li et al. (2022a)
12	Trichotomside B	Roots	CH_2_Cl_2_	95% EtOH	Hu et al. (2018)
13	Villosin A	Stems	PE	85%EtOH	Zhang et al. (2022)
14	Villosin B	Stems	EtOAc	85%EtOH	Li et al. (2014b)
15	Villosin C	Leaves	PE	95%EtOH	Xu et al. (2015)
		Stems	EtOAc	85%EtOH	Li et al. (2014b)
		Stems	*n*-BuOH	85%EtOH	Li et al. (2022b)
		Roots	PE/EtOAc (1:1, v/v)		Wang et al. (2013a)
16	6-Methoxyvillosin C	Roots	PE/EtOAc (1:1, v/v)		Wang et al. (2013a)
17	18-Hydroxy-6-methoxyvillosin C	Roots	PE/EtOAc (1:1, v/v)		Wang et al. (2013a)
18	(10*R*,16*S*)-12,16-epoxy-11,14-dihydroxy-6-methoxy17 (15→16)-abeo-abieta-5,8,11,13-tetraene-3,7-dione	Roots	PE/EtOAc (1:1, v/v)		Wang et al. (2013a)
19	Teuvinceone A	Stems	EtOAc	85%EtOH	Li et al. (2014a)
20	Teuvinceone B	Stems	EtOAc	85%EtOH	Li et al. (2014a)
		Stems	*n*-BuOH	85%EtOH	Li et al. (2022b)
21	12,16-Epoxy-11,14-dihydroxy-6-methoxy-17 (15→16)-abeo-abieta-5,8,11,13, 15-pentaene-3,7-dione	Roots	PE/EtOAc (1:1, v/v)		Wang et al. (2013a)
22	Teuvinceone H	Stems	EtOAc	85%EtOH	Li et al. (2014a)
23	15,16-Dehydroteuvincenone G	Roots	CH_2_Cl_2_	95% EtOH	Hu et al. (2018)
24	3-Dihydroteuvincenone G	Roots	CH_2_Cl_2_	95% EtOH	Hu et al. (2018)
25	17-Hydroxymandarone B	Roots	CH_2_Cl_2_	95% EtOH	Hu et al. (2018)
26	Uncinatone	Stems	EtOAc	85%EtOH	Li et al. (2014a)
		Stems	*n*-BuOH	85%EtOH	Li et al. (2022b)
		Roots	PE/EtOAc (1:1, v/v)		Wang et al. (2013a)
		Fruits	PE	95%EtOH	Wang et al. (2021)
27	Mandarone E	Roots	PE/EtOAc (1:1, v/v)		Wang et al. (2013a)
28	Formidiol	Roots	PE/EtOAc (1:1, v/v)		Wang et al. (2013a)
29	Teuvincenone E	Stems	*n*-BuOH	85%EtOH	Zhang et al. (2022)
		Roots	PE/EtOAc (1:1, v/v)		Wang et al. (2013a)
30	Teuvincenone F	Stems	EtOAc	85%EtOH	Li et al. (2014a)
		Roots	PE/EtOAc (1:1, v/v)		Wang et al. (2013a)
		Fruits	PE	95%EtOH	Wang et al. (2021)
31	(10*R*,16*S*)-12,16-Epoxy-11,14-dihydroxy-18-oxo-17(15→16),18(4→3)-diabeo-abieta-3,5,8,11,13-pentaene-7-one	Roots	PE/EtOAc (1:1, v/v)		Wang et al. (2013a)
32	(10*R*,16*R*)-12,16-Epoxy11,14,17-trihydroxy-17(15→16),18(4→3)-diabeo-abieta-3,5,8,11,13-pentaene-2,7-dione	Roots	PE/EtOAc (1:1, v/v)		Wang et al. (2013a)
33	15,16-Dihydroformidiol	Roots	CH_2_Cl_2_	95% EtOH	Hu et al. (2018)
34	18-Hydroxyteuvincenone E	Roots	CH_2_Cl_2_	95% EtOH	Hu et al. (2018)
35	2*α*-Hydrocaryopincaolide F	Roots	CH_2_Cl_2_	95% EtOH	Hu et al. (2018)
36	15*α*-Hydroxyuncinatone	Roots	CH_2_Cl_2_	95% EtOH	Hu et al. (2018)
37	15*α*-Hydroxyteuvincenone E	Roots	CH_2_Cl_2_	95% EtOH	Hu et al. (2018)
38	Trichotomin B	Roots	CH_2_Cl_2_	95% EtOH	Hu et al. (2018)
39	Trichotomside A	Roots	CH_2_Cl_2_	95% EtOH.	Hu et al. (2018)
40	Szemaoenoid A	Stems	*n*-BuOH	85%EtOH	Li et al. (2022a)
41	3,12-*O*-*β*-D-Diglucopyranosyl-11,16-dihydroxyabieta-8,11,13-triene	Stems	*n*-BuOH	85%EtOH	Zhang et al. (2022)
		Stems	*n*-BuOH	85%EtOH	Li et al. (2022b)
42	Leucasinoside	Stems	*n*-BuOH	85%EtOH	Li et al. (2022a)
43	(3*S*,5*S*,10*S*,15*S*)-3*β*-[*β*-D-Glucopyranosyl-(1→6)-*β*-D-glucopyranosyl]oxy-12-(*β*-D-glucopyranosyl)oxyabieta-8,11,13-triene-11,16-diol	Stems	*n*-BuOH	85%EtOH	Li et al. (2022a)
44	(3*S*,4*R*,10*R*,16*S*)-3,4:12,16-Diepoxy-11,14-dihydroxy-17(15→16),18(4→3)-diabeo-abieta-5,8,11,13-tetraene-7-one	Roots	PE/EtOAc (1:1, v/v)		Wang et al. (2013a)
45	12,16-Epoxy-17(15→16),18(4→3)-diabeo-abieta-3,5,8,12,15-pentaene7,11,14-trione	Roots	PE/EtOAc (1:1, v/v)		Wang et al. (2013a)
46	Trichotomin A	Roots	CH_2_Cl_2_	95% EtOH	Hu et al. (2018)
47	Clerodendrin A	Leaves	Hexane/Acetone (1:2, v/v)		Nishida et al. (1989)
48	Clerodendrin B	Leaves	Hexane/Acetone (1:2, v/v)		Nishida et al. (1989)
49	Clerodendrin D	Leaves	Hexane/Acetone (1:2, v/v)		Nishida et al. (1989)
50	Clerodendrin E	Leaves	Hexane/Acetone (1:1, v/v)		Kawai et al. (1998)
51	Clerodendrin F	Leaves	Hexane/Acetone (1:1, v/v)		Kawai et al. (1998)
52	Clerodendrin G	Leaves	Hexane/Acetone (1:1, v/v)		Kawai et al. (1998)
53	Clerodendrin H	Leaves	Hexane/Acetone (1:1, v/v)		Kawai et al. (1998)
54	Clerodendrin I	Leaves	Hexane/Acetone (1:1, v/v)		Kawai et al. (1999)
55	(2*R*,3*S*,4*R*,5*R*,6*S*,9*R*,10*R*,11*S*,13*S*,16*R*)-6,19-Diacetoxy-3-[(2*R*)-2-acetoxy-2-methylbutyryloxy]-4,18:11,16:15,16-triepoxy-15-methoxy7-clerodene-2-ol	Leaves	MeOH		Ono et al. (2013)
56	Phytol	Leaves	PE	95%EtOH	Xu et al. (2015)
		Leaves	PE	95%EtOH	Hu et al. (2014)
57	Viridiol B	Leaves	PE	95%EtOH	Xu et al. (2015)
58	Trichotomone	Roots	PE/EtOAc (1:1, v/v)		Wang et al. (2013b)
Triterpenes					
59	Friedelin	Leaves	PE	95%EtOH	Hu et al. (2014)
		Stems	EtOAc	85%EtOH	Li et al. (2019)
60	Glutinol	Stems	PE	85%EtOH	Zhang et al. (2022)
61	3*β*-hydroxy-30-norlupan-20-one	Leaves	PE	95%EtOH	Xu et al. (2015)
62	Platanic acid	Stems	EtOAc	85%EtOH	Li et al. (2019)
63	Lupeol	Leaves	PE	95%EtOH	Xu et al. (2015)
		Fruits	PE	95%EtOH	Wang et al. (2021)
		Leaves	PE	95%EtOH	Hu et al. (2014)
64	Betulinic acid	Leaves	PE	95%EtOH	Xu et al. (2015)
		Fruits	PE	95%EtOH	Wang et al. (2021)
		Leaves	PE	95%EtOH	Hu et al. (2014)
		Stems	EtOAc	85%EtOH	Li et al. (2019)
65	Taraxerol	Leaves	PE	95%EtOH	Hu et al. (2014)
		Stems	EtOAc	85%EtOH	Li et al. (2019)
66	Oleanolic acid	Fruits	PE	85%EtOH	Liu et al. (2020)
67	3-*O*-Acetyl oleanolic aldehyde	Stems	PE	85%EtOH	Zhang et al. (2022)
		Fruits	PE	95%EtOH	Wang et al. (2021)
68	Ursolic aldehyde	Leaves	PE	95%EtOH	Xu et al. (2015)
		Fruits	PE	95%EtOH	Wang et al. (2021)
69	Ursolic acid	Fruits	PE	95%EtOH	Wang et al. (2021)
70	*β*-Amyrin	Stems	CH_2_Cl_2_	MeOH	Choi et al. (2012)
71	Oleanolic aldehyde	Leaves	PE	95%EtOH	Xu et al. (2015)
72	Maslinic acid	Leaves	PE	95%EtOH	Xu et al. (2015)
73	Corosolic acid	Leaves	PE	95%EtOH	Xu et al. (2015)
74	Trijugaoside A	Stems	*n*-BuOH	85%EtOH	Li et al. (2022a)
Flavonoids					
75	Apigenin	Leaves	PE	90%EtOH	Tian (2017)
		Leaves	EtOAc	Water	Yao and Guo (2010)
		Leaves and twigs	PE	95% EtOH	Xu et al. (2015)
76	Luteolin	Leaves	PE	90% EtOH	Tian (2017)
77	Chrysoeriol	Leaves	EtOAc	90% EtOH	Tian (2017)
78	Acacetin	Leaves	MeOH		Ono et al. (2013)
		Stems	EtOAc	85%EtOH	Li et al. (2019)
79	5,7-Dihydroxy3′,4′-dimethoxyflavone	Stems	EtOAc	85%EtOH	Li et al. (2019)
80	Clerodendroside	Leaves	*n*-BuOH	MeOH	Morita et al. (1977)
81	Apigenin-7-galacturonide	Leaves	MeOH		Ono et al. (2013)
82	Clerodendrin	Leaves	Calcium ion precipitates after water extraction		Zeng et al. (1963)
					Chen et al. (1988)
83	Acacetin-7-*β*-D-glucurono-*β*-(1→2)-D-glucuronide	Leaves	—		Okigawa et al. (1970)
84	Apigenin 7-*O*-*β*-D-glucuronopyranoside	Leaves	—		Min et al. (2005)
85	Apigenin-7-*O*-*β*-D-glucuronide buthyl ester	Leaves and twigs	*n*-BuOH	95%EtOH	Xu et al. (2015)
Steroids					
86	*β*-Sitosterol	Leaves	PE	95%EtOH	Hu et al. (2014)
		Stems	EtOAc	85%EtOH	Li et al. (2019)
		Fruits	PE	85%EtOH	Liu et al. (2020)
		Leaves and twigs	PE	95%EtOH	Xu et al. (2015)
87	Daucosterol	Stems	EtOAc	85%EtOH	Li et al. (2019)
		Leaves and twigs	EtOAc	95%EtOH	Xu et al. (2015)
88	Stigmasterol	Leaves	PE	95%EtOH	Hu et al. (2014)
		Stems	EtOAc	85%EtOH	Li et al. (2019)
		Fruits	PE	85%EtOH	Liu et al. (2020)
89	Stigmasterol-3-*O*-glucopyranoside	Stems	EtOAc	85%EtOH	Li et al. (2019)
90	3-*O*-*β*-D-Galactopyranosyl-(24*β*)-ethylcholesta-5, 22, 25-trien	Leaves	EtOAc	90% EtOH	Tian (2017)
91	24-Ethyl-7-oxocholesta-5, 22(*E*), 25-trien-3*β*-ol	Roots	PE/EtOAc (1:1, v/v)		Yang et al. (2014)
92	Decortinone	Roots	PE/EtOAc (1:1, v/v)		Yang et al. (2014)
93	22-Dehydroclerosterol	Leaves	PE	95%EtOH	Hu et al. (2014)
		Fruits	PE	85%EtOH	Liu et al. (2020)
		Stems	CH_2_Cl_2_	MeOH	Choi et al. (2012)
		Leaves	PE	90% EtOH	Tian (2017)
		Roots	PE/EtOAc (1:1, v/v)		Yang et al. (2014)
94	Clerosterol	Leaves	MeOH		Ono et al. (2013)
		Leaves	PE	95%EtOH	Hu et al. (2014)
		Roots	PE/EtOAc (1:1, v/v)		Yang et al. (2014)
95	(22*E*,24*R*)-Stigmasta-4,22,25-trien-3-one	Leaves	PE	95%EtOH	Xu et al. (2013)
96	(20*R*,22*E*,24*R*)-6*β*-Hydroxy-stigmasta-4,22,25-trien-3-one	Leaves	PE	95%EtOH	Xu et al. (2013)
97	(20*R*,22*E*,24*R*)-3*β*-Hydroxy-stigmasta-5,22,25-trien-7-one	Leaves	PE	95%EtOH	Xu et al. (2013)
98	(20*R*,22*E*,24*R*)-Stigmasta-5,22,25-trien-3*β*,7*β*-diol	Leaves	PE	95%EtOH	Xu et al. (2013)
99	(20*R*,22*E*,24*R*)-Stigmasta-22,25-dien-3,6-dione	Leaves	PE	95%EtOH	Xu et al. (2013)
100	(20*R*,22*E*,24*R*)-Stigmasta-22,25-dien-3*β*,6*β*,9*α*-triol	Leaves	EtOAc	95%EtOH	Xu et al. (2013)
101	22-Dehydroclerosterol 3*β*-*O*-*β*-D-(6′-*O*-margaroyl)-glucopyranoside	Leaves	PE	95%EtOH	Xu et al. (2013)
Phenylpropanoids					
102	Acteoside (Verbascoside)	Flowers	EtOAc	MeOH	Lee et al. (2016)
		Leaves	MeOH		Ono et al. (2013)
		Leaves and twigs	*n*-BuOH	95%EtOH	Xu et al. (2015)
		Stems	EtOAc	MeOH	Kang et al. (2003)
		Stems	EtOAc	MeOH	Kim et al. (2001)
		Leaves	Water	70% MeOH	Lee J. Y. et al. (2011)
		Stems	*n*-BuOH	85%EtOH	Zhang et al. (2022)
		Leaves	Water	70% MeOH	Kim et al. (2009)
103	Martynoside	Flowers	EtOAc	MeOH	Lee et al. (2016)
		Stems	*n*-BuOH	85%EtOH	Li et al. (2022b)
		Leaves and twigs	*n*-BuOH	95%EtOH	Xu et al. (2015)
		Stems	EtOAc	MeOH	Kang et al. (2003)
		Stems	EtOAc	MeOH	Kim et al. (2001)
104	Leucosceptoside A	Flowers	EtOAc	MeOH	Lee et al. (2016)
		Stems	EtOAc	MeOH	Kang et al. (2003)
		Stems	EtOAc	MeOH	Kim et al. (2001)
105	Jionoside D	Stems	EtOAc	MeOH	Kim et al. (2001)
		Stems	EtOAc	MeOH	Chae et al. (2004)
106	2″-Acetylmartynoside	Stems	CH_2_Cl_2_	MeOH	Chae et al. (2007)
107	3″-Acetylmartynoside	Stems	CH_2_Cl_2_	MeOH	Chae et al. (2007)
108	Isoacteoside (Isoverbascoside)	Flowers	EtOAc	MeOH	Lee et al. (2016)
		Stems	EtOAc	MeOH	Kang et al. (2003)
		Stems	EtOAc	MeOH	Kim et al. (2001)
		Leaves	Water	70% MeOH	Kim et al. (2009)
		Stems	EtOAc	MeOH	Chae et al. (2005)
		Stems	*n*-BuOH	85%EtOH	Zhang et al. (2022)
109	Isomartynoside	Stems	*n*-BuOH	85%EtOH	Zhang et al. (2022)
		Stems	*n*-BuOH	85%EtOH	Li et al. (2022b)
		Stems	EtOAc	MeOH	Kang et al. (2003)
		Stems	EtOAc	MeOH	Kim et al. (2001)
110	Plantainoside C	Stems	EtOAc	MeOH	Kim et al. (2001)
111	*p*-Hydroxyphenethyl-*trans*-ferulate	Fruits	EtOAc	85%EtOH	Liu et al. (2020)
112	Syringaresinol	Stems	*n*-BuOH	85%EtOH	Li et al. (2022b)
113	Syringaresinol-4ʹ-*O*-*β*-glucopyranoside	Stems	*n*-BuOH	85%EtOH	Li et al. (2022b)
114	Cistanoside F	Stems	*n*-BuOH	85%EtOH	Li et al. (2022b)
115	Glypentoside C	Stems	*n*-BuOH	85%EtOH	Li et al. (2022b)
116	Cannabisin E	Fruits	EtOAc	85%EtOH	Liu et al. (2020)
117	Cannabisin F	Fruits	EtOAc	85%EtOH	Liu et al. (2020)
118	Grossamide	Fruits	EtOAc	85%EtOH	Liu et al. (2020)
119	*N*-*p*-Coumaryltyramine	Fruits	EtOAc	85%EtOH	Liu et al. (2020)
120	Spicatolignan B	Stems	EtOAc	85%EtOH	Li et al. (2014a)
121	Lyoniresinol	Stems	*n*-BuOH	85%EtOH	Zhang et al. (2022)
122	Lyonirenisol-3*α*-*O*-*β*-D-glucopyranoside	Stems	*n*-BuOH	85%EtOH	Zhang et al. (2022)
123	Ecdysanols D	Leaves and twigs	PE	95%EtOH	Gao et al. (2022)
124	Ecdysanols E	Leaves and twigs	PE	95%EtOH	Gao et al. (2022)
125	Trichotomoside	Stems	CH_2_Cl_2_	MeOH	Chae et al. (2006)
Phenylethanosides					
126	Decaffeoylverbascoside	Stems	*n*-BuOH	85%EtOH	Zhang et al. (2022)
		Leaves	Water	70% MeOH	Kim et al. (2009)
127	2-(4-Hydroxyphenyl) ethanol-*O*-*β*-D-glucopyranosyl-(1→2)-*O*-*β*-D-glucopyranoside	Stems	*n*-BuOH	85%EtOH	Li et al. (2022b)
128	Deacylisomartynoside	Stems	*n*-BuOH	85%EtOH	Zhang et al. (2022)
Phenolic glycosides					
129	3,4-Dimethoxyphenyl-1-*O*-*β*-D-apiofuranosyl (1→2)-*β*-D-glucopyranoside	Stems	*n*-BuOH	85%EtOH	Li et al. (2022b)
130	2,6-Dimethoxy-4-hydroxy-1-*O*-*β*-D-glucopyranoside	Stems	*n*-BuOH	85%EtOH	Li et al. (2022b)
Anthraquinones					
131	Aloe-emodin	Stems	EtOAc	85%EtOH	Li et al. (2014a)
132	Emodin	Stems	EtOAc	85%EtOH	Li et al. (2014a)
133	Chrysophanol	Stems	EtOAc	85%EtOH	Li et al. (2014a)
Polyketones					
134	Clerodendruketone A	Leaves and twigs	PE	95%EtOH	Gao et al. (2022)
135	Clerodendruketone B	Leaves and twigs	PE	95%EtOH	Gao et al. (2022)
Cyclohexylethanoids					
136	Rengyolone	Leaves	PE	95%EtOH	Xu et al. (2014)
137	5-*O*-Butyl cleroindin D	Leaves	PE	95%EtOH	Xu et al. (2014)
138	Cleroindin C	Leaves	PE	95%EtOH	Xu et al. (2014)
139	1-Hydroxy-1-(8-palmitoyloxyethyl) cyclohexanone	Leaves	PE	95%EtOH	Xu et al. (2014)
140	Cleroindin B	Leaves	PE	95%EtOH	Xu et al. (2014)
141	Rengyol	Leaves	PE	95%EtOH	Xu et al. (2014)
142	Isorengyol	Leaves	PE	95%EtOH	Xu et al. (2014)
Alkaloids					
143	Trichotomine	Fruits	EtOH		Iwadare et al. (1974)
144	Trichotomine G_1_	Fruits	EtOH		Iwadare et al. (1974)
145	1H-Indole-3-carboxylic acid	Leaves	PE	95%EtOH	Hu et al. (2014)
146	Orixine	—			Xu and Xing (1962)
147	Orixidine	—			Xu and Xing (1962)
148	Iso-orixine	—			Xu and Xing (1962)
149	1H-Imidazole-4-carboxylate	Fruits	PE	95%EtOH	Wang et al. (2021)
Acid amides					
150	(2*S*,3*S*,4*R*,*E*)- 2-[(2′*R*)- 2′-Hydroxyl-octacosane amino-23-hexacosane-1,3,4-triol	Leaves	EtOAc	90% EtOH	Tian (2017)
151	Aurantiamide acetate	Leaves and twigs	PE	95%EtOH	Xu et al. (2015)
Acids					
152	Palmitic acid	Fruits	PE	85%EtOH	Liu et al. (2020)
		Leaves	PE	90% EtOH	Tian (2017)
		Leaves	PE	Water	Yao and Guo (2010)
153	(2S,3S,4R,23E)-2-[(2′R)-2′-hydroxyl-octacosane amino]-hexacosane-1,3,4-triol	Leaves	PE	Water	Yao and Guo (2010)
154	Corchorifalty acid E	Leaves and twigs	EtOAc	95%EtOH	Xu et al. (2015)
Alcohols					
155	*n*-Decanol	Leaves and twigs	PE	95%EtOH	Xu et al. (2015)
156	*n*-Heptadecanol	Leaves	PE	90%EtOH	Tian (2017)
157	*α*-Tocopherol	Leaves	PE	95%EtOH	Xu et al. (2015)
Aldehydes					
158	Isovanillin	Stems	EtOAc	85%EtOH	Li et al. (2019)
159	Syringaldehyde	Stems	EtOAc	85%EtOH	Li et al. (2019)
160	5-Hydroxymethylfurfural	Leaves and twigs	PE	95%EtOH	Xu et al. (2015)
Esters					
161	Dibutyl phthalate	Stems	EtOAc	85%EtOH	Li et al. (2019)
162	Monopalmitin	Leaves and twigs	PE	95%EtOH	Xu et al. (2015)
Alkane					
163	*n*-Pentatriacontane	Leaves	EtOAc	90% EtOH	Tian (2017)
Peroxide					
164	Bungein A	Fruits	EtOAc	85%EtOH	Liu et al. (2020)

### 5.1 Terpenoids

**FIGURE 4:**
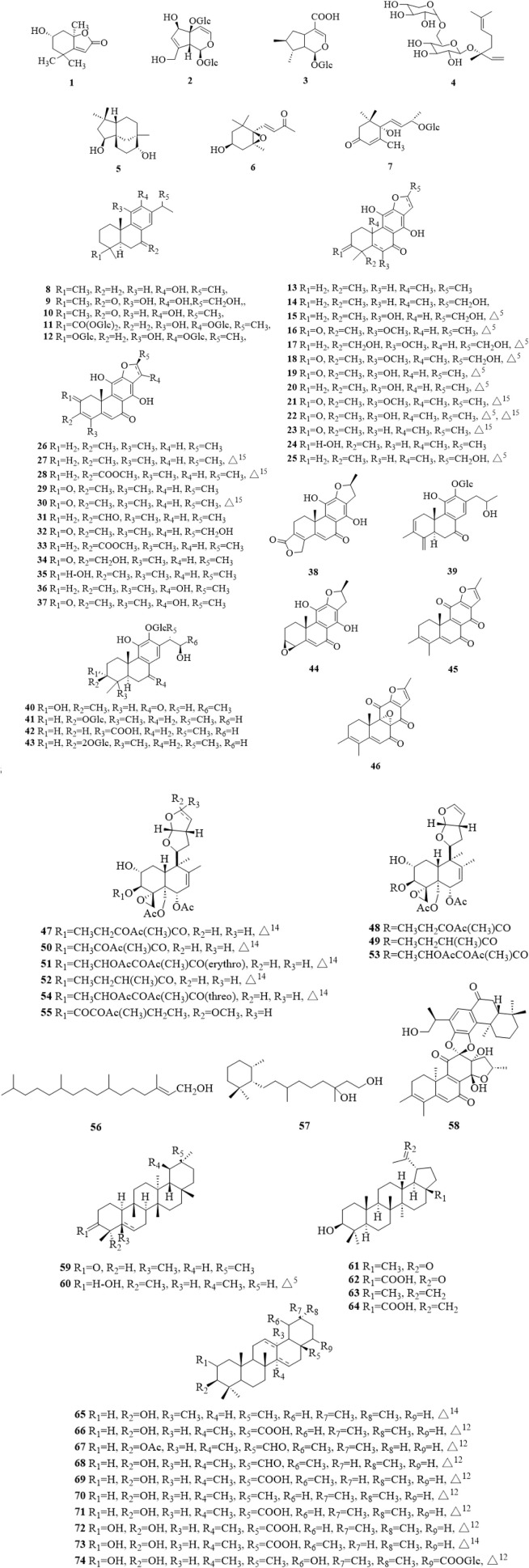
The chemical structure of terpenoids **1–74** isolated from *Clerodendrum trichotomum*.

Terpenoids are the most commonly reported types of compounds isolated from *C. trichotomum*, with a total of 74 compounds (Figure 4), including monoterpenes (**1–4**) (Nagao et al., 2001; Lee et al., 2016; Li et al., 2022a), sesquiterpenes (**5–7**) (Xu et al., 2015), diterpenes (**8–58**) and triterpenoids (**59–74**) (Choi et al., 2012; Hu et al., 2014; Xu et al., 2015; Li et al., 2019; Liu et al., 2020; Wang et al., 2021; Li et al., 2022a; Zhang et al., 2022). Among the diterpenes, there were 39 abietane-type diterpenes (**8–46**) (Wang et al., 2013a; Li et al., 2014a; Li et al., 2014b; Xu et al., 2015; Hu et al., 2018; Wang et al., 2021; Li et al., 2022a; Li et al., 2022b; Zhang et al., 2022), 9 clerodane-type diterpene (**47–55**) (Nishida et al., 1989; Kawai et al., 1998; Kawai et al., 1999; Ono et al., 2013), a chained diterpene (**56**) (Hu et al., 2014; Xu et al., 2015), a monocyclic diterpene (**57**) (Xu et al., 2015) and a dimeric diterpene (**58**) (Wang et al., 2013b). Abietane-type diterpenes are also the most abundant of all isolated secondary metabolites. Due to the limitation of science and technology at that time, there were some errors in some literatures. Li et al. discovered and revised the NMR data and chemical system names of some compounds in the literatures (Li et al., 2022a; Li et al., 2014b).

### 5.2 Flavonoids

**FIGURE 5:**
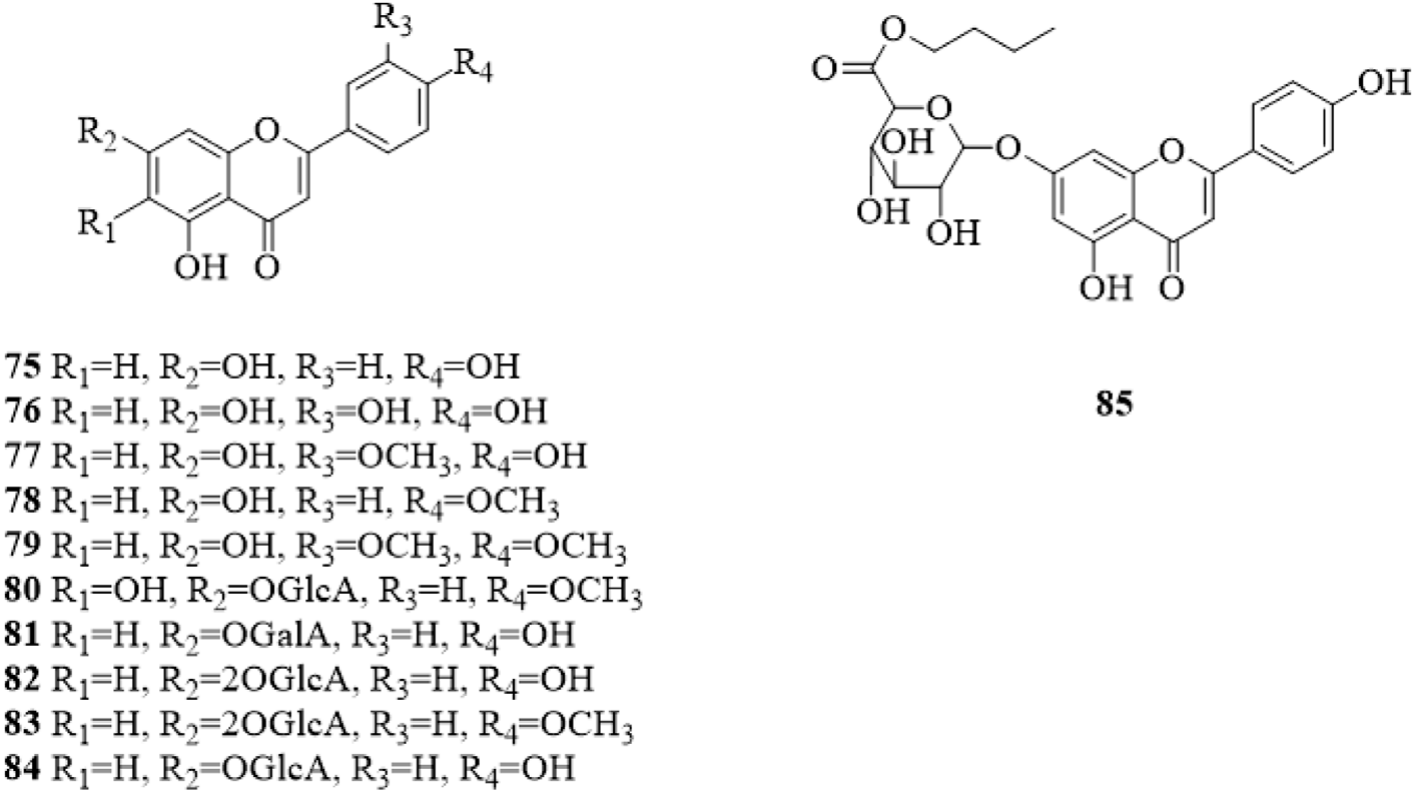
The chemical structure of flavonoids **75–85** isolated from *Clerodendrum trichotomum*.

Flavonoids are mainly found in the leaves of *C. trichotomum*. Up to now, only 11 flavonoids (**75**–**85**, Figure 5) (Zeng et al., 1963; Okigawa et al., 1970; Morita et al., 1977; Chen et al., 1988; Min et al., 2005; Yao and Guo, 2010; Ono et al., 2013; Xu and Shi, 2015; Tian, 2017; Li et al., 2019) have been isolated from *C. trichotomum*. The only difference is the substituents in the 6, 7, 3′ and 4′ positions of the flavonoid, the linked sugar group is mostly glucuronic acid (GlcA).

### 5.3 Steroids

**FIGURE 6:**
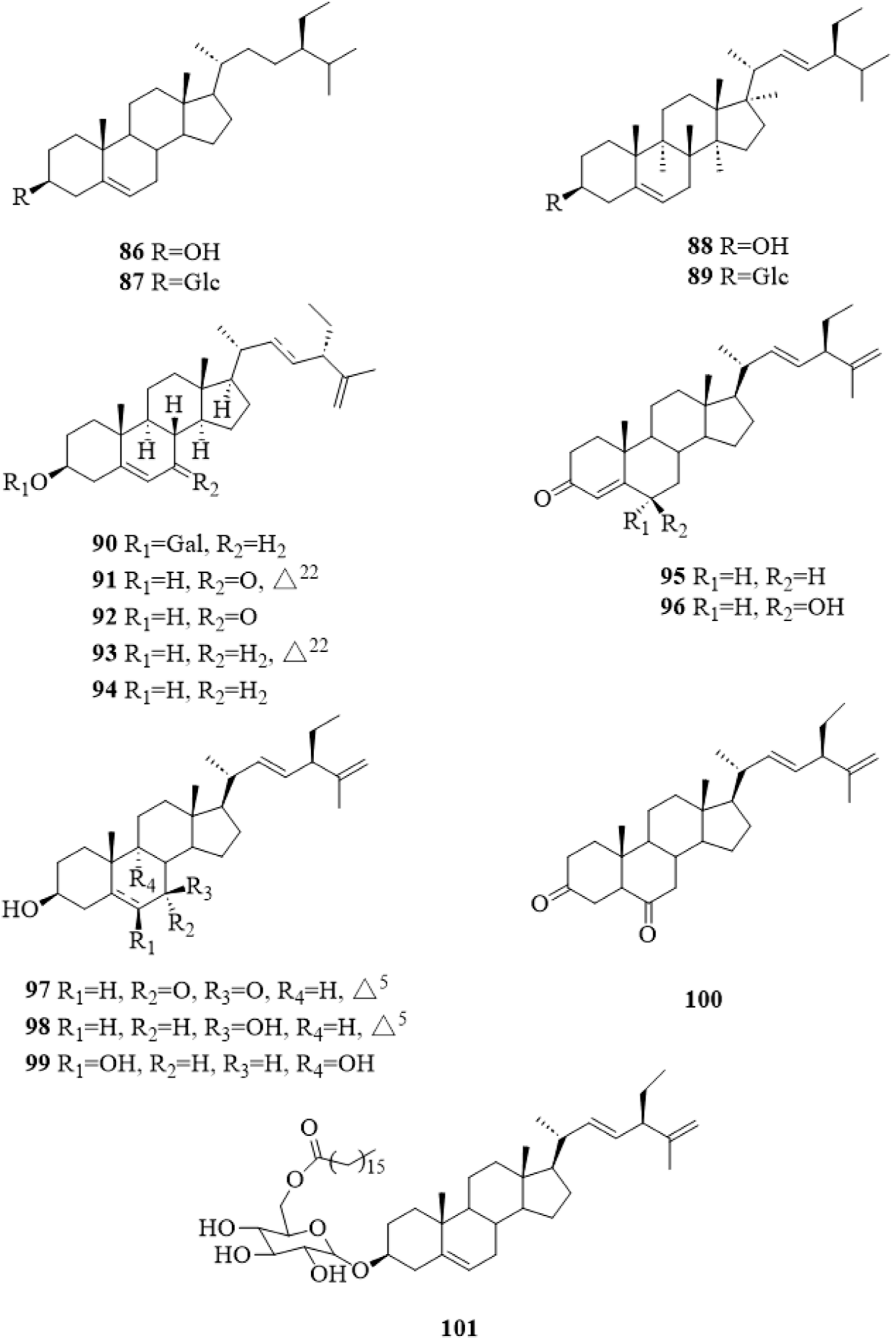
The chemical structure of steroids **86–101** isolated from *Clerodendrum trichotomum*.

There were 16 steroid compounds (**86**–**101**, Figure 6) (Choi et al., 2012; Ono et al., 2013; Xu et al., 2013; Hu et al., 2014; Yang et al., 2014; Xu and Shi, 2015; Tian, 2017; Li et al., 2019; Liu et al., 2020) isolated from *C. trichotomum*. Interestingly, there are nine steroids with a side chain of C_10_H_17_ and two double bonds at C-22 and C-25.

### 5.4 Phenylpropanoids

**FIGURE 7:**
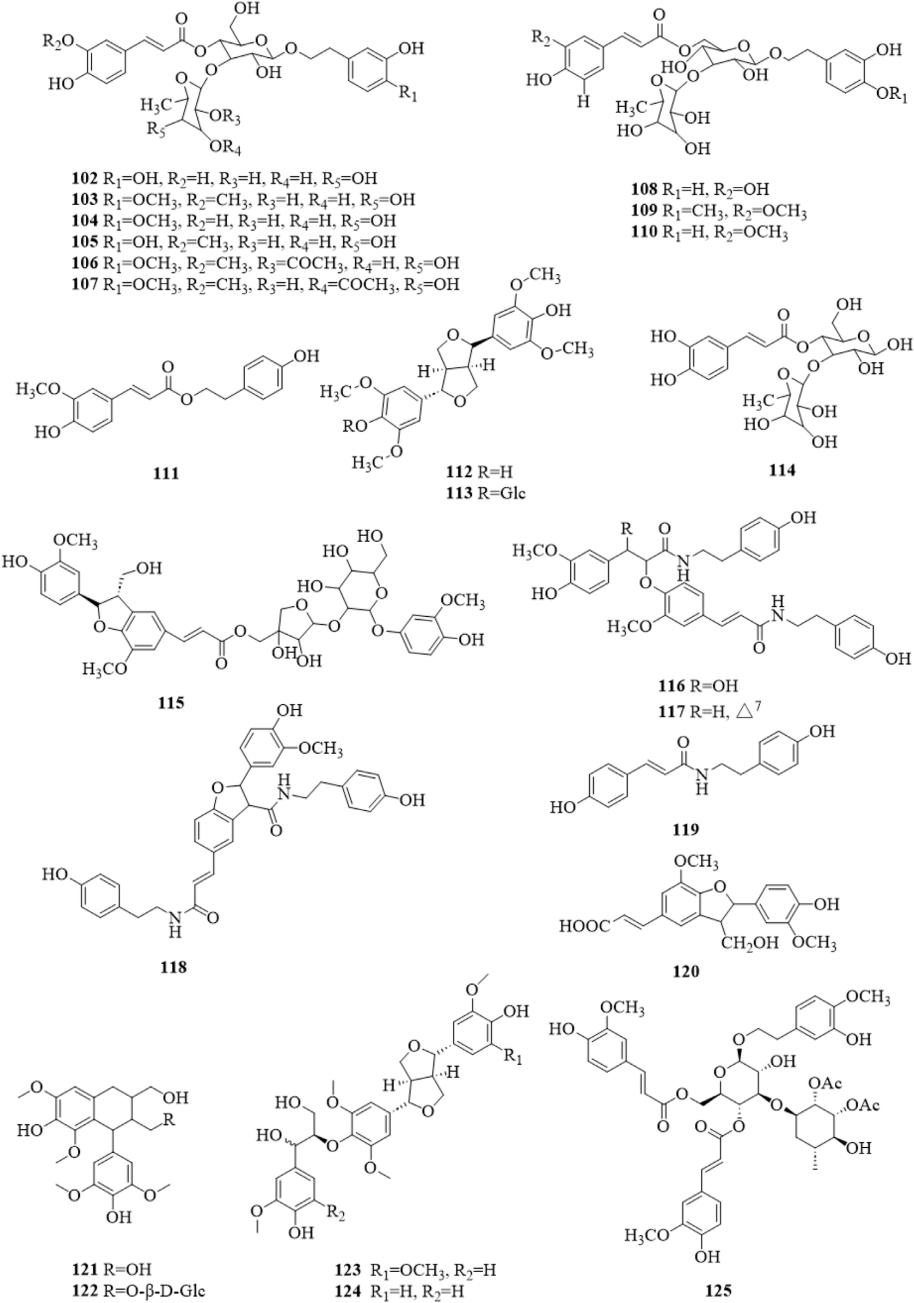
The chemical structure of phenylpropanoids **102–125** isolated from *Clerodendrum trichotomum*.

There were 24 phenylpropanoid compounds (**102–125**, Figure 7) reported in the literature (Kim et al., 2001; Kang et al., 2003; Chae et al., 2004; Chae et al., 2005; Chae et al., 2006; Chae et al., 2007; Kim et al., 2009; Lee D. G. et al., 2011; Ono et al., 2013; Li et al., 2014a; Xu and Shi, 2015; Lee et al., 2016; Liu et al., 2020; Gao et al., 2022; Li et al., 2022b; Zhang et al., 2022). The isolated phenylpropanoids were second only to terpenoids in quantity. They are more polar, mostly in the form of glycosides, and often contain caffeoyl groups in the molecule.

### 5.5 Phenylethanosides

**FIGURE 8:**
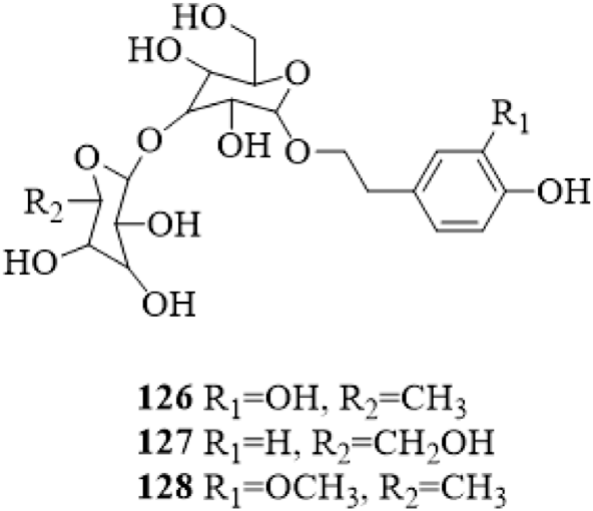
The chemical structure of phenylethanosides **126–128** isolated from *Clerodendrum trichotomum*.

Three phenylethanosides, decaffeoylverbascoside (**126**), 2-(4-hydroxyphenyl) ethanol-*O*-*β*-D-glucopyranosyl-(1→2)-*O*-*β*-D-glucopyranoside (**127**) and deacylisomartynoside (**128**) were isolated from the stems of *C. trichotomum* (Zhang et al., 2022; Li et al., 2022b). Compound **126** was also yielded from the leaves (Kim et al., 2009). The structure of compounds **126**–**128** is shown in Figure 8.

### 5.6 Phenolic glycosides

**FIGURE 9:**
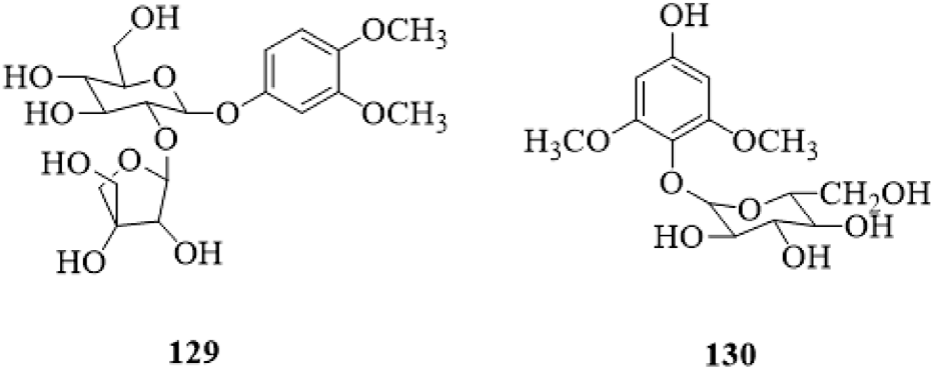
|The chemical structure of phenolic glycosides **129**,**130** isolated from *Clerodendrum trichotomum*.

3,4-dimethoxyphenyl-1-*O*-*β*-D-apiofuranosyl (1→2)-*β*-D-glucopyranoside (**129**) and 2,6-dimethoxy-4-hydroxy-1-*O*-*β*-D-glucopyranoside (**130**) have been isolated from the stems, and both of them are phenolic glycosides (Li et al., 2022b). The structures are shown in Figure 9.

### 5.7 Anthraquinones

**FIGURE 10:**
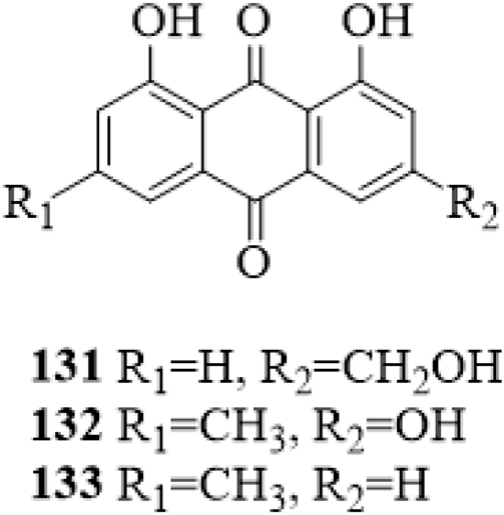
The chemical structure of anthraquinones **131–133** isolated from *Clerodendrum trichotomum*.

Three anthraquinones (**131**–**133**, Figure 10) were isolated from the stems of *C*. *trichotomum* (Li et al., 2014a). They were reported from the genus *Clerodendrum* for the first time.

### 5.8 Polyketones

**FIGURE 11:**
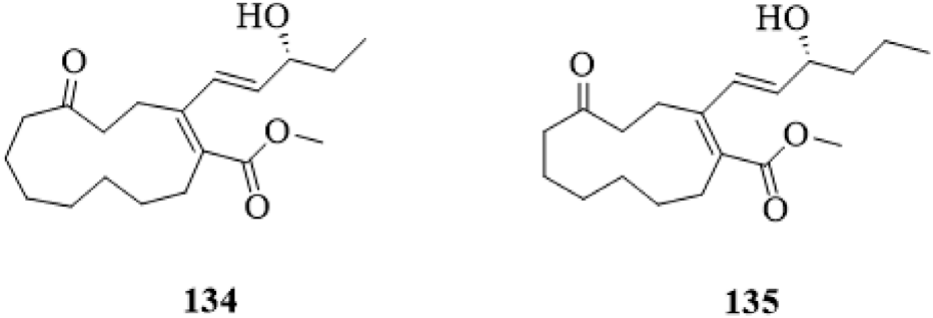
The chemical structure of polyketones **134**,**135** isolated from *Clerodendrum trichotomum*.

Two polyketones (Figure 11), Clerodendruketone A (**134**) and Clerodendruketone B (**135**), from the leaves and twigs of *C*. *trichotomum* were reported (Gao et al., 2022).

### 5.9 Cyclohexylethanoids

**FIGURE 12:**
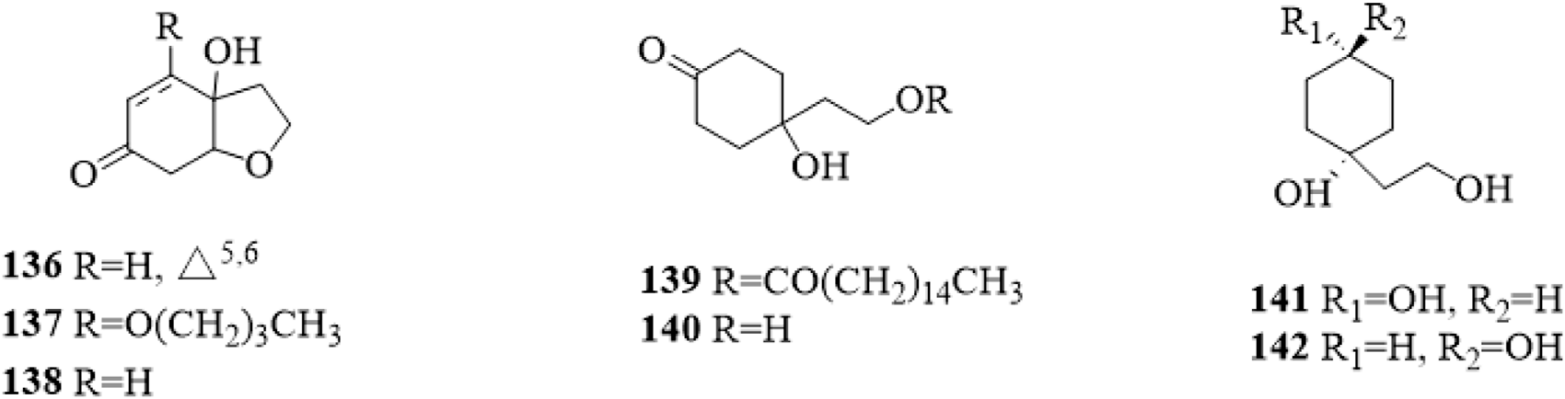
The chemical structure of cyclohexylethanoids **136–142** isolated from *Clerodendrum trichotomum*.

Cyclohexylethanoids are rarely isolated from *C*. *trichotomum*. In 2014, 7 cyclohexylethanoids (Figure 12), including rengyolone (**136**), 5-*O*-butyl cleroindin D (**137**), cleroindin C (**138**), 1-hydroxy-1-(8-palmitoyloxyethyl) cyclohexanone (**139**), cleroindin B (**140**), rengyol (**141**) and isorengyol (**142**), were isolated from the leaves and reported (Xu et al., 2014).

### 5.10 Alkaloids

**FIGURE 13:**
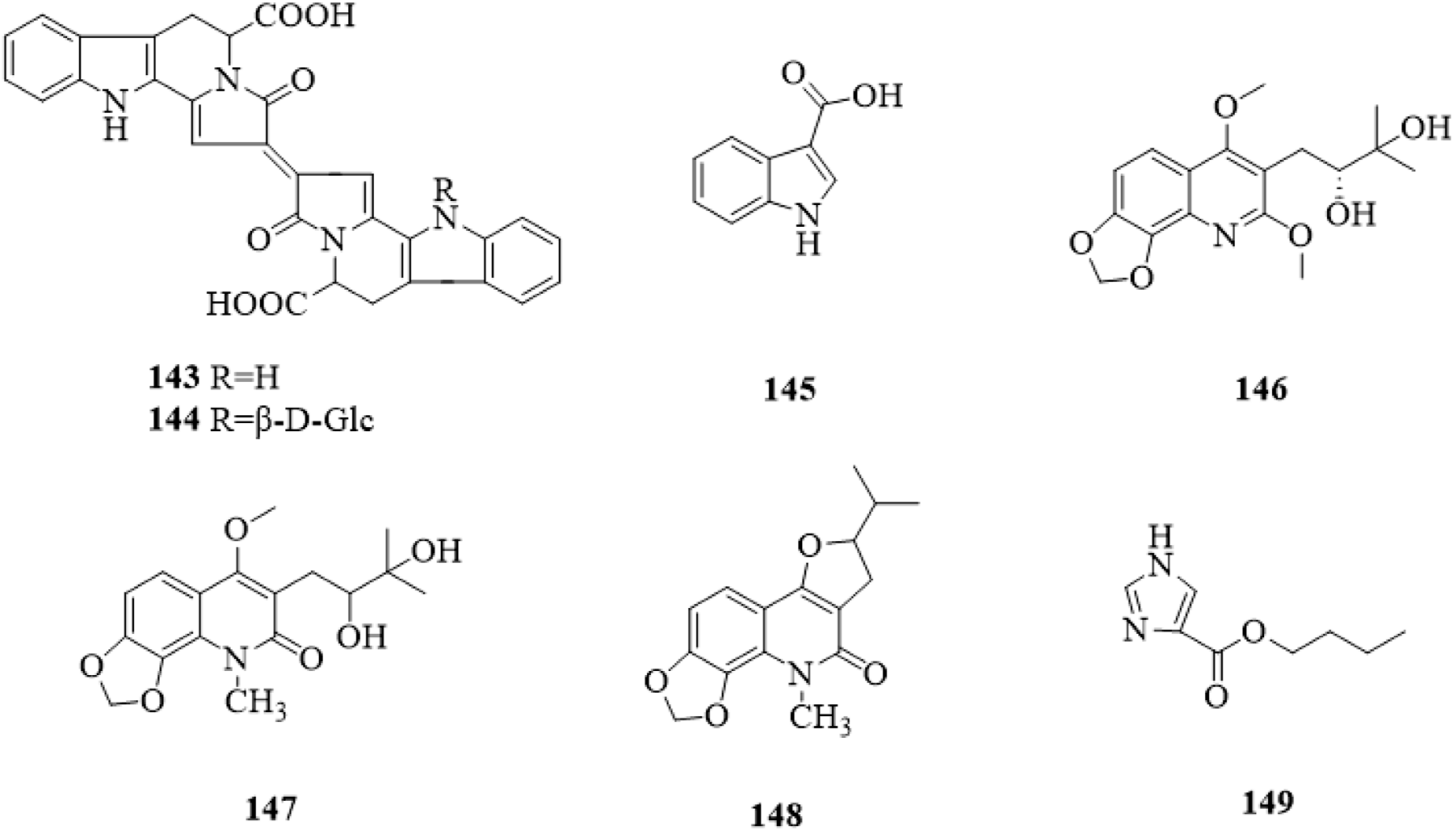
The chemical structure of alkaloids **143–149** isolated from *Clerodendrum trichotomum*.

So far, a total of 8 alkaloids (**143**–**149**, Figure 13) have been found from *C*. *trichotomum*. In the 1960s, Xu et al. isolated three quinoline alkaloids (Xu and Xing, 1962), namely, orixine (**146**), orixidine (**147**) and iso-orixine (**148**). Then, 1H-indole-3-carboxylic acid (**145**) (Hu et al., 2014), trichotomine (**143**) and trichotomine G_1_ (**144**) (Iwadare et al., 1974) have been discovered, which were all indole alkaloids. In recent years, 1H-imidazole-4-carboxylate (**149**) (Wang et al., 2021) were reported, and they belong to purine and imidazole, respectively.

### 5.11 Acid amides

**FIGURE 14:**
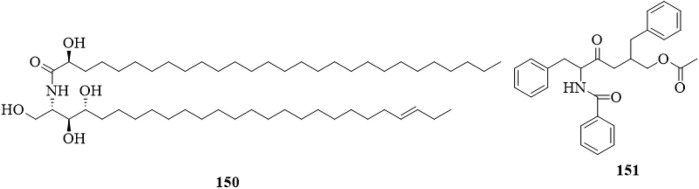
The chemical structure of acid amides **150**,**151** isolated from *Clerodendrum trichotomum*.

Two acid amides, including (2S,3S,4R,23E)-2-[(2′R)-2′-hydroxyl-octacosane amino]-hexacosane-1,3,4-triol (**150**) (Tian, 2017) and aurantiamide acetate (**151**) (Xu and Shi, 2015) have been isolated and reported. The structures of the two compounds are shown in Figure 14.

### 5.12 Others

**FIGURE 15:**
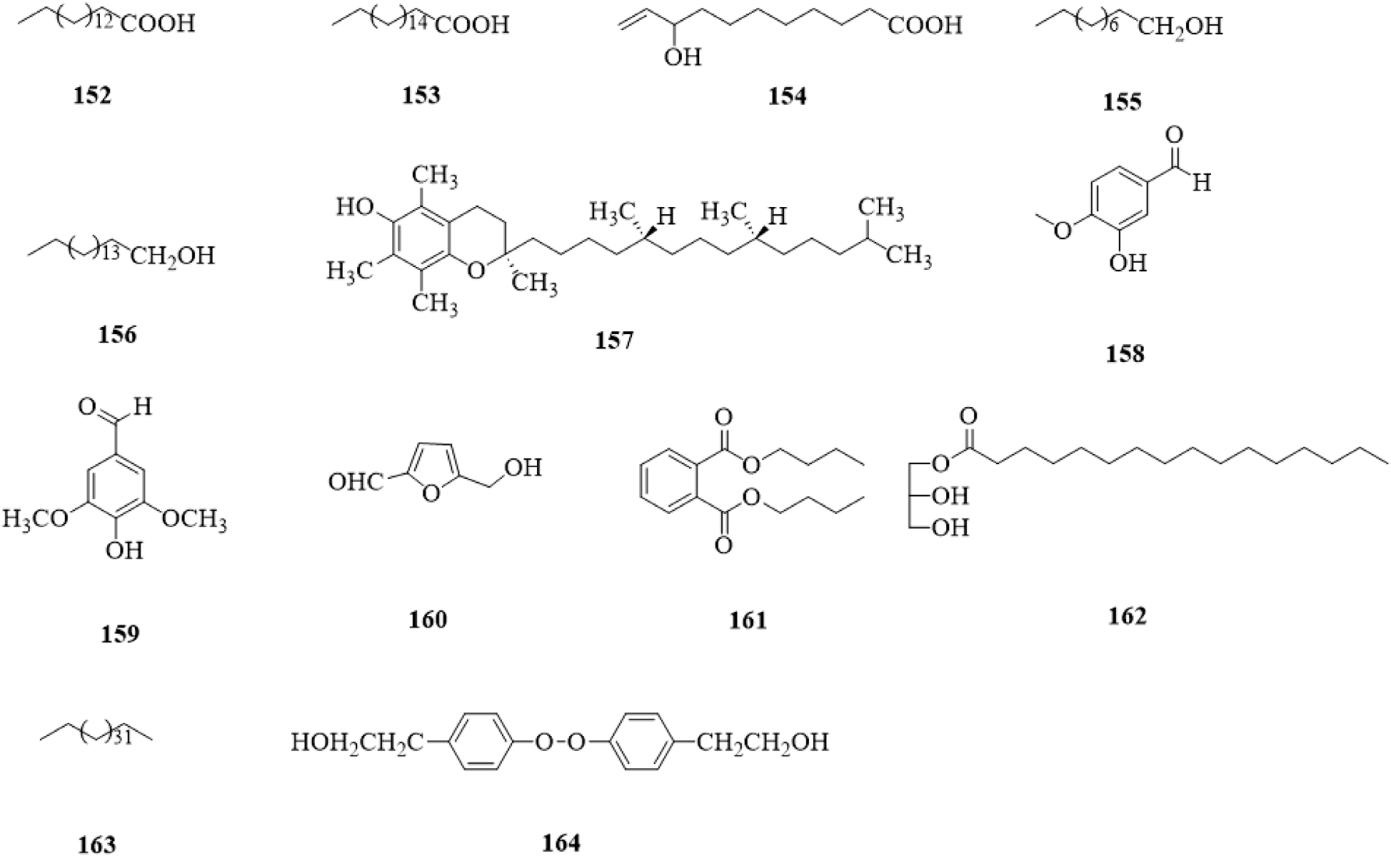
The chemical structure of other compounds **152–164** isolated from *Clerodendrum trichotomum*.

Other types of compounds (**152**–**164**, Figure 15) include acids (**152–154**) (Yao and Guo, 2010; Xu and Shi, 2015; Tian, 2017; Liu et al., 2020), alcohols (**155–157**) (Xu et al., 2015; Xu and Shi, 2015; Tian, 2017), aldehydes (**158–160**) (Xu and Shi, 2015; Li et al., 2019), esters (**161,162**) (Xu and Shi, 2015; Li et al., 2019), a alkane (**163**) (Tian, 2017), and a peroxide (**164**) (Liu et al., 2020).

### 5.13 Volatile compounds

Volatile compounds of *C*. *trichotomum* have been analyzed and reported by gas chromatography-mass spectrometry (GC-MS), and most of these studies were focused on the leaves, flowers and fruits (Table 3). Yan et al. extracted volatile oil from the leaves of *C*. *trichotomum* by steam distillation, analyzed and identified 47 compounds (Yan and Tian, 2003). Among them, (*E*,*E*,*E*)-9,12,15-octadecatrienoate methyl ester and (*E*,*E*,E) −9,12,15-octadecatrienol accounted for the higher content, accounting for 12.65% and 13.4% of the total components respectively. Lee et al. used GC-MS to analyse the volatile components of the leaves, and identified 50 compounds (Lee J. Y. et al., 2011), of which the main components were 2,6,10,15-tetramethylheptadecane (23.83%) and linalool (29%). The volatiles of the flowers were analyzed by headspace solid phase microextraction coupled with GC-MS (HS-SPME-GC-MS) for the first time (Tian et al., 2011). Twenty-seven compounds were identified, 2,6,10,14-tetramethylhexadecane, hexadecanal, octen-3-ol, benzaldehyde and *n*-hexadecanoic acid were the dominant components. Li et al. used GC-MS to analyse the volatile oil components in the fruits of *C*. *trichotomum* in 2020, and identified a total of 31 compounds (Li et al., 2020). The analysis showed that the components of volatile oil were different with different parts of plant, origin and extraction methods.

**TABLE 3:** Volatile compounds from *Clerodendrum trichotomum.*

Source	Method	Chief components (≧4%)	References
Leaves	GC-MS	(*E*,*E*,*E*)-9,12,15-Octadecatrien-1-ol (13.4%) (*E*,*E*,*E*)-9,12,15-Octadecatrienoic acid methyl ester (12.65%) Palmitic acid (12.51%) *n*-Pentadecanoic acid (7.66%)	Yan and Tian (2003)
		2,6,10,15-Tetramethylheptadecane (23.83%) Linalool (29%)	Lee D. G. et al. (2011)
Flowers	HS-SPME-GC-MS	2,6,10,14-Tetramethylhexadecane (17.25%) Hexadecanal (10.57%) 1- Octen-3-ol (6.78%) Benzaldehyde (6.10%) *n*-Hexadecanoic acid (4.85%)	Tian et al. (2011)
Fruits	GC-MS	Dotriacontane (7.117%) Tetratriacontane (7.111%) Hentriacontane (6.141%) Tritriacontane (5.753%) Pentatriacontane (5.107%) Triacontane (4.53%) Nonacosane (4.092%)	Li et al. (2020)

## 6 Pharmacological activities

*C. trichotomum* is widely used in the folk to nourish the liver and reduce blood pressure, dispell wind and eliminate dampness. Pharmacological studies have shown that it has a variety of pharmacological activities. In China, relevant studies mainly focus on the mechanism of reducing blood pressure, sedation and analgesia, most of which were in the 1950s and 1960s. Studies in other countries mainly concentrated in antiinflammatory, antioxidant and other mechanisms of action, with more reports in South Korea and Japan, and most of the studies were carried out in the 21st century. The chemical constituents isolated from the *C. trichotomum* have abundant pharmacological activities, the most important ones are antihypertensive, antitumour, antioxidant, antiinflammatory, antibacterial, sedative and analgesic. The pharmacological activities of all the compounds are shown in Table 4.

**TABLE 4:** Pharmacological activities of compounds isolated from *Clerodendrum trichotomum*.

Activities	Compounds /Extracts	Types	Testing subjects	Dose/Duration	Controls	Effects/Mechanisms	References
Anti-hypertensive activity							
	Water extracts	*In vivo*	Rats/dogs	50–100 mg/kg for 2 weeks	NM	Showed hypotensive effect on anesthetized or awake rats and dogs, renal hypertensive rats and dogs	Xu and Xing (1962)
		*In vivo*	Rats/dogs	Rats: 2.5 mL/100 g for 3 h; Dogs: 0.2 g/kg for 30 min	NM	The antihypertensive effects are closely related to the central nervous and endovascular receptors.	Xu and Peng (1960)
		*In vivo*	Dogs/rats/cats /rabbits	Dogs/rabbits/cats: 0.5–1 g/kg; Rats: 25 g/kg, 10 g/kg	NM	The mechanism of antihypertensive action is related to the direct dilation of blood vessels and the blocking of ganglia.	Xu (1962)
		*In vivo*	Rats/dogs	Rats: 0.1 g/kg 0.5 g/kg; Dogs: 0.24 g/kg	NM	Reduces blood pressure by increasing renal blood flow and promoting urinary water and sodium excretion.	Lu et al. (1994)
	Acteoside (**102**)	*In vitro*	ACE inhibitory activity	10 μL for 15 min	NM	IC_50_ value was 373.3 ± 9.3 μg/mL	Kang et al. (2003)
	Martynoside (**103**)	*In vitro*	ACE inhibitory activity	10 μL for 15 min	NM	IC_50_ value was 524.4 ± 28.1 μg/mL	Kang et al. (2003)
	leucosceptoside A (**104**)	*In vitro*	ACE inhibitory activity	10 μL for 15 min	NM	IC_50_ value was 423.7 ± 18.8 μg/mL	Kang et al. (2003)
	Isoacteoside (**108**)	*In vitro*	ACE inhibitory activity	10 μL for 15 min	NM	IC_50_ value was 376.0 ± 15.6 μg/mL	Kang et al. (2003)
	Isomartynoside (**109**)	*In vitro*	ACE inhibitory activity	10 μL for 15 min	NM	IC_50_ value was 505.9 ± 26.7 μg/mL	Kang et al. (2003)
	Clerodendroside (**82**)	*In vivo*	Rats	50 mg/kg	NM	Have a hypotensive effect on anaesthetised rats	Morita et al. (1977)
Antitumour activity							
	Cyrtophyllone B (**9**)	*In vitro*	Human cancer cell lines (A549, HepG-2, MCF-7 and 4T1)	0.15, 0.5, 1.5, 5, 15, 50 μM for 2 d	Doxorubicin	IC_50_ values against 4 tumor cells were 14.96, 15.00, >50, and 35.46 μM, respectively	Li et al. (2014b)
	Villosin B (**14**)	*In vitro*	Human cancer cell lines (A549, HepG-2, MCF-7 and 4T1)	0.15, 0.5, 1.5, 5, 15, 50 μM for 2 d	Doxorubicin	IC_50_ values against 4 tumor cells were 15.00, 29.74, >50, and >50 μM, respectively	Li et al. (2014b)
	Villosin C (**15**)	*In vitro*	Human cancer cell lines (A549, HepG-2, MCF-7 and 4T1)	0.15, 0.5, 1.5, 5, 15, 50 μM for 2 d	Doxorubicin	IC_50_ values against 4 tumor cells were 14.93, 31.35, >50, and >50 μM, respectively	Li et al. (2014b)
		*In vitro*	Human cancer cell lines (K562, MCF-7, A549 and HepG2)	0, 2, 4, 8, 16, 32, 64 μM for 2 d	Doxorubicin	IC_50_ values against 4 tumor cells were 28.41, >100, >100 and 31.35 μM, respectively	Li et al. (2022b)
	Teuvincenone A (**19**)	*In vitro*	Human cancer cell lines (A549, HepG-2, MCF-7, 4T1)	0.15, 0.5, 1.5, 5, 15, 50 μM for 2 d	Doxorubicin	IC_50_ values against 4 tumor cells were >50, >50, 25.00, and 17.83 μM, respectively	Li et al. (2014b)
	Teuvincenone B (**20**)	*In vitro*	Human cancer cell lines (K562, MCF-7, A549 and HepG2)	0, 2, 4, 8, 16, 32, 64 μM for 2 d	Doxorubicin	IC_50_ values against 4 tumor cells were >100, 43.18, >100, and 29.74 μM, respectively	Li et al. (2022b)
	Uncinatone (**26**)	*In vitro*	Human cancer cell lines (A549, HepG-2, MCF-7 and 4T1)	0.15, 0.5, 1.5, 5, 15, 50 μM for 2 d	Doxorubicin	IC_50_ values against 4 tumor cells were >50, 12.50, >50, and 8.79 μM, respectively	Li et al. (2014b)
		*In vitro*	Human cancer cell lines (K562, MCF-7, A549 and HepG2)	0, 2, 4, 8, 16, 32, 64 μM for 2 d	Doxorubicin	IC50 values against 4 tumor cells were >100, 25.00, 22.34, and 12.50 μM, respectively	Li et al. (2022b)
		*In vitro*	Human cancer cell lines (BGC-823, Huh-7, KB, KE-97 and Jurkat)	72 h	Staurosporine	IC_50_ values against 5 tumor cells were 26.47, 43.88, 19.90, 32.94 and 17.95 μM, respectively	Wang et al. (2013a)
	Mandarone E (**27**)	*In vitro*	Human cancer cell lines (BGC-823, Huh-7, KB, KE-97 and Jurkat)	72 h	Staurosporine	IC_50_ values against 5 tumor cells were 31.95, 50.99, 16.05, 32.69 and 16.83 μM, respectively	Wang et al. (2013a)
	Teuvincenone E (**29**)	*In vitro*	Human cancer cell lines (BGC-823, Huh-7, KB, KE-97 and Jurkat)	72 h	Staurosporine	IC_50_ values against 5 tumor cells were 3.95, 5.37, 1.18, 1.27 and 0.83μM, respectively	Wang et al. (2013a)
	(10*R*,16*S*)-12,16-Epoxy-11,14-dihydroxy-18-oxo-17(15→16),18(4→3)-diabeo-abieta-3,5,8,11,13-pentaene-7-one (**31**)	*In vitro*	Human cancer cell lines (BGC-823, Huh-7, KB, KE-97 and Jurkat)	72 h	Staurosporine	IC_50_ values against 5 tumor cells were 15.72, 29.33, 7.50, 10.39 and 8.58 μM, respectively	Wang et al. (2013a)
	(3*S*,4*R*,10*R*,16*S*)-3,4:12,16-Diepoxy-11,14-dihydroxy-17(15→16),18(4→3)-diabeo-abieta-5,8,11,13-tetraene-7-one (**44**)	*In vitro*	Human cancer cell lines (BGC-823, Huh-7, KB, KE-97 and Jurkat)	72 h	Staurosporine	IC_50_ values against 5 tumor cells were 8.02, 12.60, 5.45, 7.01 and 3.49μM, respectively	Wang et al. (2013a)
	12,16-Epoxy-17(15→16),18(4→3)-diabeo-abieta-3,5,8,12,15-pentaene7,11,14-trione (**45**)	*In vitro*	Human cancer cell lines (BGC-823, Huh-7, KB, KE-97 and Jurkat)	72 h	Staurosporine	IC_50_ values against 5 tumor cells were 19.48, 29.41, 12.26, 28.19 and 18.10 μM, respectively	Wang et al. (2013a)
	Trichotomone (**58**)	*In vitro*	Human cancer cell lines (BGC-823, Huh-7, KB, KE-97 and Jurkat)	NM	Staurosporine	IC_50_ values against 4 tumor cells were 9.80, 19.38, 9.42, 7.51 μM, respectively	Wang et al. (2013b)
	(20*R*,22*E*,24*R*)-3*β*-Hydroxy-stigmasta-5,22,25-trien-7-one (**97**)	*In vitro*	HeLa cell line	24 h	NM	IC_50_ value against Hela cell was 35.67 μM	Xu et al. (2014)
	(20*R*,22*E*,24*R*)-Stigmasta-5,22,25-trien-3*β*,7*β*-diol (**98**)	*In vitro*	HeLa cell line	24 h	NM	IC_50_ value against Hela cell was 28.92 μM	Xu et al. (2014)
	Acteoside (**102**)	*In vitro*	Human cancer cell lines (MK-1, HeLa and B16F10)	50 μL for 48 h	NM	GI_50_ values against 3 tumor cellswere 40, 66, 8 μM, respectively	Nagao et al. (2001)
	Isoacteoside (**108**)	*In vitro*	Human cancer cell lines (MK-1, HeLa and B16F10)	50 μL for 48 h	NM	GI_50_ values against 3 tumor cells were 40, 61, 8 μM, respectively	Nagao et al. (2001)
Antioxidant activity							
	Acteoside (**102**)	*In vitro*	DPPH, H_2_O_2_, O_2_^−^ and NO	DPPH: 10 μL for 30 min; H_2_O_2_: 5 μL for 10 min; O_2_^−^: 10 μL for 10 min; NO: 5 μL for 150 min	Trolox and curcumin	EC_50_ values were 22.2, 80.2, 23.4 μM in the scavenging of DPPH radical, H_2_O_2_ and O_2_ radical.	Ono et al. (2013)
			DPPH	5, 10, 50, 100 μg/mL	L-Ascorbic acid	Scavenging effect were 92.29, 93.05, 92.61, 101.13% at 5, 10, 50, 100 μg/mL, respectively	Lee et al. (2016)
			DPPH ROS	DPPH: 20 µL for 30 min	DPPH: Ascorbate	DPPH scavenging effect were 9, 30, 78% at 0.1, 1, 10 μM, respectively. ROS generation were 200, 190, 150% at 1, 3, 10 μM, respectively.	Yoon et al. (2009)
	Martynoside (**103**)	*In vitro*	DPPH	5, 10, 50, 100 μg/mL	L-Ascorbic acid	Scavenging effect were 11.00, 38.82, 72.80, 85.66% at 5, 10, 50, 100 μg/mL, respectively	Lee et al. (2016)
	Leucosceptoside A (**104**)	*In vitro*	DPPH	5, 10, 50, 100 μg/mL	L-Ascorbic acid	Scavenging effect were 35.47, 46.53, 95.95, 86.33% at 5, 10, 50, 100 μg/mL, respectively	Lee et al. (2016)
	Jionoside D (**105**)	*In vitro*	ROS DPPH	30 min 5 h	N-acetylcystein	Intracellular ROS scavenging activity activities were 41% (0.1 mg/mL), 62% (1 mg/mL) and 85% (10 mg/mL), respectively. DPPH radical scavenging activity was 25% at 0.1 mg/mL, 36% at 1 mg/mL, and 55% at 10 mg/mL	Chae et al. (2004)
	2″-Acetylmartynoside (**106**)	*In vitro*	ROS DPPH	ROS:10 μg/mL for 30 min; DPPH:10 μg/mL for 5 h	Negative: Glucose Positive: *N*-acetylcysteine	ROS scavenging activity is 55.3% in 10 ug/mL. DPPH scavenging activity is 32.3% in 10 ug/mL	Chae et al. (2007)
	3″-Acetylmartynoside (**107**)	*In vitro*	ROS DPPH	ROS:10 μg/mL for 30 min; DPPH:10 μg/mL for 5 h	Negative: Glucose Positive: *N*-acetylcysteine	ROS scavenging activity is 58.5% in 10 ug/mL. DPPH scavenging activity is 36.1% in 10 ug/mL	Chae et al. (2007)
	Isoacteoside (**108**)	*In vitro*	DPPH	5, 10, 50, 100 μg/mL	L-Ascorbic acid	Scavenging effect were 90.94, 91.56, 91.87, 100.05% at 5, 10, 50, 100 μg/mL, respectively	Lee et al. (2016)
			DPPH ROS	ROS: 0.1, 1, 10 μg/mL for 30 min; DDPH: 0.1, 1, 10 μg/mL for 5 h	*N*-acetylcysteine	ROS scavenging activity showed concentration dependence: 43% at 0.1 μg/mL, 68% at 1 μg/mL, and 83% at 10 μg/mL. DPPH radical scavenging activity and had significance only at 10 μg/mL.	Chae et al. (2005)
			DPPH ROS	DPPH: 20 µL for 30 min	DPPH: Ascorbate	DPPH scavenging effect were 2, 10, 67% at 0.1, 1, 10 μM, respectively. ROS generation were 250, 230, 200% at 1, 3, 10 μM, respectively.	Yoon et al. (2009)
	Ecdysanols D (**123**)	*In vitro*	DPPH	NM	Vitamin C	IC_50_ value was 66.07 ± 13.29 µM	Gao et al. (2022)
	Ecdysanols E (**124**)	*In vitro*	DPPH	NM	Vitamin C	IC_50_ value was 53.60 ± 6.68 µM	Gao et al. (2022)
	Trichotomoside (**125**)	*In vitro*	DPPH ROS	5, 10, 20 and 40 μM for 48 h	Epigallocatechin gallate	The DPPH radical scavenging activity was 12, 20, 36, and 59% in 5, 10, 20, and 40 μM, respectively. The ROS-scavenging activity was 39, 49, 58% and 61% in 5, 10, 20, and 40 μM, respectively.	Chae et al. (2006)
	Apigenin 7-galacturonide (**81**)	*In vitro*	DPPH, H_2_O_2_, O_2_^−^ and NO	DPPH: 10 μL for 30 min; H_2_O_2_: 5 μL for 10 min; O_2_^−^: 10 μL for 10 min; NO: 5 μL for 150 min	Trolox and curcumin	EC_50_ value against NO-scavenging activity was 452 μM.	Ono et al. (2013)
Antiinflammatory activity							
	Methanol extracts	*In Vivo* /*in vitro*	Rats, mice and Raw 264.7 cells	1 mg/kg	Indomethacin	The anti-inflammatory activity of 60% methanol extract was higher than indomethacin, and it also inhibited the production of PGE_2_ in RAW 264.7 cells	Choi et al. (2004)
		*In vitro*	LPS-induced RAW 264.7 cells	0.1, 0.5, 1 mg/mL	NM	Iinhibited the expression of the pro-inflammation gene through the inhibition of NF-κB dependent pathway in RAW 264.7 cells.	Park and Kim (2007)
	Villosin C (**15**)	*In vitro*	NO	NM	Aminoguanidine hydrochloride	IC_50_ value against NO production was 15.5 μM	Hu et al. (2018)
	15,16-Dehydroteuvincenone G (**23**)	*In vitro*	NO	NM	Aminoguanidine hydrochloride	IC_50_ value against NO production was 6.0 μM	Hu et al. (2018)
	2*α*-Hydrocaryopincaolide F (**35**)	*In vitro*	NO	NM	Aminoguanidine hydrochloride	IC_50_ value against NO production was 6.5 μM	Hu et al. (2018)
	15α-Hydroxyteuvincenone E (**37**)	*In vitro*	NO	NM	Aminoguanidine hydrochloride	IC_50_ value against NO production was 38.6 μM	Hu et al. (2018)
	Trichotomin B (**38**)	*In vitro*	NO	NM	Aminoguanidine hydrochloride	IC_50_ value against NO production was 28.9 μM	Hu et al. (2018)
	Trichotomin A (**46**)	*In vitro*	NO	NM	Aminoguanidine hydrochloride	IC_50_ value against NO production was 10.6 μM	Hu et al. (2018)
	80% Methanol	*In vivo*	Mice/rats	Mice: 1 mg/kg; Rats: 10 mg/kg for 4 h	Indomethacin	The maximal inhibitory activity was 47.0% in the vascular permeability test. Inhibition rate of rat paw oedema was 59.5% at 1 h	Kim et al. (2009)
	Acteoside (**102**)	*In vivo*	Mice/rats	Mice: 1 mg/kg; Rats: 10 mg/kg for 4 h	Indomethacin	The maximal inhibitory activity was 46.5% in the vascular permeability test. Inhibition rate of rat paw oedema was 63.82% at 1 h	Kim et al. (2009)
		*In vitro*	Histamine, arachidonic acid release, PGE_2_, PLA_2_	Histamine: 10 min; PLA_2_: 20 min	NM	Inhibition of histamine release induced by melittin, arachidonic acid, thapsigargin, possibly related to extracellular Ca^2+^	Lee et al. (2006)
		*In vitro*	Histamine, Phospholipase A_2_	NM	NM	Inhibited protein content at a dose of 25 mg/kg, and histamine content and PLA_2_ activity at a dose of 50 mg/kg	Lee D. G. et al. (2011)
	Apigenin-7-*O*-*β*-D-glucuronopyranoside (**84**)	*In vivo*	Rats	0.01–30 mg/kg	omeprazole	IC_50_ values were 0.2, 0.1, 0.2 mg/kg in reflux oesophagitis in rats, NSAID-induced gastritis, gastric secretion in reflux oesophagitis, respectively	Min et al. (2005)
	24-Ethyl-7-oxocholesta-5,22(*E*),25-trien-3*β*-ol (**91**)	*In vitro*	HT-29 cells	0–50 μg/mL for 24 h	Blank: IL-1β timulation alone	At 118, 59, and 30 μmol/L, the production of IL-8 was reduced to 20.8%, 40.0%, and 59.2% of the control level, respectively.	Yang et al. (2014)
Antibacterial activity							
	*n*-Hexane, MC, ethyl acetate And *n*-butanol fractions	*In vitro*	*S. aureus*, *E*. *coli* and *H*. *pylori*	1.7 mg/mL for 24 h	Penicillin	The *n*-hexane and MC fractions showed antibacterial activity against *H. pylori* and showed inhibition zones of 10 and 11 mm in disc assay, respectively.	Choi et al. (2012)
	*β*-Amyrin (**70**)	*In vitro*	*S. aureus*, *E*. *coli* and *H*. *pylori*	3.4 mg/mL for 24 h	Penicillin	Clear inhibition zones were 12, 10, and 13 mm, respectively.	Choi et al. (2012)
	22-Dehydroclerosterol (**93**)	*In vitro*	*S. aureus*, *E*. *coli* and *H*. *pylori*	3.4 mg/mL for 24 h	Penicillin	Clear inhibition zones were 11, 11, and 9 mm, respectively.	Choi et al. (2012)
	Clerodendruketone A (**134**)	*In vitro*	*E*. *Coli* and *S*. *aureus*	50 μg/mL	Cefalexin, Levofloxacin and Vancomycin	Bacteriostatic rates against *E*. *coli*, *S*. *aureus* were 30%–60%, 60%–80%, respectively	Gao et al. (2022)
	Clerodendruketone B (**135**)	*In vitro*	*E*. *coli* and *S*. *aureus*	50 μg/mL	Cefalexin, Levofloxacin and Vancomycin	Bacteriostatic rates against *E. coli* and *S*. *aureus* were 30%–60%	Gao et al. (2022)
Analgesic effect							
	Decoction	*In vivo*	Mice	1.65, 6.6, 16.5 g/kg for 120 min	Morphine and antipyrine	The peak value appeared 20–40 min after administration, then gradually decreased, and could be maintained for 2 h	Wang and Shen (1957)
	Clerodendronin B	*In vivo*	Mice	4, 8 mg/10 g for 2 h	Morphine	The analgesic percentage (50%–80%) was stronger than that of morphine (30%–60%)	Xu et al. (1960b)
Sedative effect							
	Decoction	*In vivo*	Mice	0.2, 0.4, 0.6, 0.8, 1.0, 1.2 mL for 24 h	NM	Have a mild sedative effect on mice, and increased doses did not induce sleep	Shen and Wang (1957)
	Clerodendronin A	*In vivo*	Mice	5, 10 mg/20 g for 2 h	Chlorpromazine, rifampicin	Sedative index (5.58) was stronger than the effect of rifampicin (3.38)	Xu et al. (1960a)
Anti HIV-1 activity							
	Acteoside (**102**)	*In vitro*	HIV-1 integrase	90 min	Curcumin and L-chicoric acid	IC_50_ value against HIV-1 integrase was 7.8 μM	Kim et al. (2001)
	Isoacteoside (**108**)	*In vitro*	HIV-1 integrase	90 min	Curcumin and L-chicoric acid	IC_50_ value against HIV-1 integrase was 13.7 μM	Kim et al. (2001)
Whitening activity							
	Acteoside (**102**)	*In vitro*	Tyrosinase activity	100 µM for 10 min	Arbutin	Inhibition ratios of tyrosinase activity were 102.9, 104.9, 107.7% at 1, 10, 100 µM.	Yoon et al. (2009)
	Isoacteoside (**108**)	*In vitro*	Tyrosinase activity	100 µM for 10 min	Arbutin	Inhibition ratios of tyrosinase activity were 98.1, 100.3, 115.7% at 1, 10, 100 µM.	Yoon et al. (2009)
Insect feeding stimulant activity							
	Clerodendrin B (**48**)	*In vivo*	Turnip sawfly	0.1–10 μg/64 mm^2^	NM	100% feeding response at 10 μg/64 mm^2^	Nishida et al. (1989)
		*In vivo*	Turnip sawfly	NM	NM	Effective dose was 2 μg/grain	Nishida and Fukami (1990)
	Clerodendrin D (**49**)	*In vivo*	Turnip sawfly	0.1–10 μg/64 mm^2^	NM	At 10 μg/64 mm^2^, the feeding response is close to 100%	Nishida et al. (1989)
		*In vivo*	Turnip sawfly	NM	NM	Effective dose was 2 μg/grain	Nishida and Fukami (1990)
	Clerodendrin H (**53**)	*In vivo*	Turnip sawfly	10^–11^–10^–6^/filter paper	NM	100% feeding response at 10^−7^g/64 mm^2^	Kawai et al. (1998)
	Clerodendrin I (**54**)	NM					Kawai et al. (1999)
Inhibition of *Eichhornia crassipes* activity							
	Extracts	*In vitro*	Chlorophyll content, catalase activity and malondialdehyde content	0.04 g/mL for 18 d	Blank: sodium phosphate buffer; Control: glycerol	Chlorophyll content was 0.710 mg/g, peroxidase activity 67.500 U/g/min, malondialdehyde content 12.015 mmol/g.	Zheng and Lu (2012)

^a^ Note: NM, not mentioned; ACE, angiotensin converting enzyme; MC, methylene chloride; *S. aureus, Staphylococcus aureus; E. coli, Escherichia coli; H. pylor, Helicobacter pylori*.

### 6.1 Antihypertensive activity

The study on the antihypertensive pharmacology of *C. trichotomum* first began in China in the 1950s and 1960s. The water extracts of the leaves and twigs of *C. trichotomum* (“Chou Wu Tong” in Chinese) showed different degrees of hypotensive effect on anesthetized or awake rats and dogs, as well as renal hypertensive rats and dogs, regardless of oral administration or injection (Xu and Xing, 1962). Xu and Peng reported that the antihypertensive effects are closely related to the central nervous and endovascular receptors (Xu and Peng, 1960). Other research have shown that its antihypertensive mechanism is closely related to the direct dilation of blood vessels and the blocking of ganglia (Xu, 1962). Lu et al. found that *C. trichotomum* is effective in lowering blood pressure by increasing renal blood flow and promoting urinary water and sodium excretion (Lu et al., 1994).

Kang et al. isolated a series of phenylpropanoid glycosides from the stems of *C*. *trichotomum* and found that these compounds acteoside (**102**), martynoside (**103**), leucosceptoside A (**104**), isoacteoside (**108**), and isomartynoside (**109**) had significant angiotensin converting enzyme (ACE) inhibitory activity, with IC_50_ values were 373.3 ± 9.3 µg/mL, 524.4 ± 28.1 µg/mL, 423.7 ± 18.8 µg/mL, 376.0 ± 15.6 µg/mL, 505.9 ± 26.7 µg/mL, respectively. The antihypertensive effect of *C. trichotomum* may be, at least in part, due to ACE inhibitory effect of the phenylpropanoid glycosides (Kang et al., 2003).

Morita et al. isolated a flavonoid glycoside, clerodendroside (**82**), from the leaves of *C*. *trichotomum*, which proved to have a hypotensive effect on anaesthetised rats (Morita et al., 1977).

### 6.2 Antitumour activity

The large number of compounds in *C. trichotomum* has a variety of anti-tumour activities, including breast cancer cells MCF-7, 4T1, lung cancer cells A549, hepatocellular carcinoma cells HepG2, cervical cancer cells Hela, melanoma cells B16, haematological leukemia cells K562, acute lymphoblastic leukemia cells CEM, acute promyelocytic leukaemia cells HL-60, intestinal cancer cells HCT-8 and so on.

Li et al. isolated nine abietane diterpenoids from the EtOAc part of the stems of *C. trichotomum,* and cyrtophyllone B (**9**), villosin B (**14**), villosin C (**15**), teuvincenone A (**19**), uncinatone (**26**) were found to have remarkable cytotoxicity against four cancer cell lines (A549, HepG-2, MCF-7 and 4T1) with IC_50_ values ranging from 8.79 to 35.46 µM (Li et al., 2014b). Then, 4 abietane diterpenoids were obtained from the n-butanol portion by the same research group, and villosin C (**15**), teuvincenone B (**20**) and ucinatcone (**26**) were found to have good antitumour activity (Li et al., 2022b). Ucinatcone (**26**) inhibited the proliferation of MCF-7, A549 and HepG2 cells most strongly, with the IC_50_ of 25.00, 22.34, and 12.50 μM, respectively. Only villosin C (**15**) had inhibitory activity on the proliferation of K562 cells, with an IC_50_ of 28.41 μM. Teuvincenone B (**20**) had some inhibitory activity on the proliferation of MCF-7 and HepG2 cells, with IC_50_ of 43.18 and 29.74 μM, respectively. Wang et al. isolated and characterized 14 rearranged abietane diterpenoids from the roots of *C. trichotomum* (Wang et al., 2013a). All isolates were tested for their cytotoxicities against five human cancer cell lines (BGC-823, Huh-7, KB, KE-97, and Jurkat), only uncinatone (**26**), mandarone E (**27**), teuvincenone E (**29**), (10R,16S)-12,16-epoxy-11,14-dihydroxy-18-oxo-17(15→16),18(4→3)-diabeo-abieta-3,5,8,11,13-pentaene-7-one (**31**), (3S,4R,10R,16S)-3,4:12,16-diepoxy-11,14-dihydroxy-17(15→16),18(4→3)-diabeo-abieta-5,8,11,13-tetraene-7-one (**44**) and 12,16-epoxy-17(15→16),18(4→3)-diabeo-abieta-3,5,8,12,15-pentaene7,11,14-trione (**45**) were found to show cytotoxic effects with IC_50_ values of 0.83–50.99 μM. The study of structure-activity relationship (SAR) shows that the rearranged A-ring and an intact 2-methyl-2-dihydrofuran moiety are supposed to be necessary to demonstrate cytotoxicity. A dimeric diterpene trichotomone (**58**) was isolated from the roots and inhibited *in vitro* cytotoxicities against several human cancer cell lines (A549, Jurkat, BGC-823 and 293TWT) with IC_50_ values ranging from 7.51 to 19.38 μM (Wang et al., 2013b).

Five steroids were isolated from the leaves of *C. trichotomum* (Xu et al., 2013), of which (20*R*,22*E*,24*R*)-3*β*-hydroxy-stigmasta-5,22,25-trien-7-one (**97**) and (20*R*,22*E*,24*R*)-stigmasta-5,22,25-trien-3*β*,7*β*-diol (**98**) exhibited moderate cytotoxicity *in vitro* against HeLa cell line, with IC_50_ at 35.67 and 28.92 μg/mL, respectively. The same research group (Xu et al., 2014) also isolated seven cyclohexylethanoids from the leaves. 5-*O*-butyl cleroindin D (**137**) and 1-hydroxy-1-(8-palmitoyloxyethyl) cyclohexanone (**139**) were evaluated for their cytotoxicity against A549 human tumor cell line, but there were no obvious cytotoxicity.

Nagao et al. regarded acteoside (**102**) and isoacteoside (**108**) as the antiproliferative constituents and found that the content of active compounds was higher in the bark of *C. trichotomum* (ca. 4.6%) than in leaves (ca. 0.6%). The GI_50_ of acteoside (**102**) against three tumor cell lines (MK-1: human gastric adenocarcinoma, HeLa: human uterus carcinoma, and B16F10: murine melanoma) were 40, 66 and 8 μM, while GI_50_ of isoacteoside (**108**) were 40, 61, 8 μM, respectively. SAR study suggests that the antiproliferative activities of phenylpropanoids depend on the 3,4-dihydroxyphenethyl group with some contribution of the 3,4-dihydroxycinnamoyl (caffeoyl) group (Nagao et al., 2001).

### 6.3 Antioxidant activity

Phenylpropanoid glycosides 2″-acetylmartynoside (**106**) and 3″-acetylmartynoside (**107**), isolated from the stems of *C*. *trichotomum*, showed antioxidant activity in terms of intracellular reactive oxygen species (ROS) scavenging effects (Chae et al., 2007). Jionoside D (**105**), isoacteoside (**108**) and trichotomoside (**125**), also isolated from the stems, exhibited scavenging activity of intracellular ROS and of 1,1-diphenyl-2-picrylhydrazyl (DPPH) radical, as well as lipid peroxidation inhibitory activity. This radical scavenging activity of them protected the cell viability of Chinese hamster lung fibroblast (V79-4) cells exposed H_2_O_2_ (Chae et al., 2004; Chae et al., 2005; Chae et al., 2006). Furthermore, jionoside D (**105**) and isoacteoside (**108**) increased the activities of cellular antioxidant enzymes, superoxide dismutase (SOD) and catalase (CAT) (Chae et al., 2004; Chae et al., 2005). Gao et al. isolated ecdysanols D (**123**) and ecdysanols E (**124**) from the leaves and twigs, which had moderate antioxidant activity with respective IC_50_ values of 66.07 and 53.60 μM (Gao et al., 2022). Lee et al. isolated five compounds from the flowers and found that acteoside (**102**), martynoside (**103**), leucosceptoside A (**104**), isoacteoside (**108**) exhibited strong DPPH antioxidant activity (Lee et al., 2016). Yoon et al. isolated acteoside (**102**) and isoacteoside (**108**) from *C. trichotomum*, both of which dose-dependently inhibited silica-induced ROS production in B16 melanoma cells (Yoon et al., 2009). Ono et al. also reported that among six isolated compounds, acteoside (102) showed the strongest activity with EC_50_ values of 22.2, 80.2, 23.4 μM in the scavenging of DPPH radical, H_2_O_2_ and O_2_^−^ radical (Ono et al., 2013). Taken together, these findings suggest that phenylpropanoid glycosides isolated from *C. trichotomum* exhibits antioxidant properties.

A flavonoid glycoside apigenin 7-galacturonide (**81**) was isolated from the leaves of *C. trichotomum* and exhibited moderate NO scavenging activity, with the EC_50_ 452 μM (Ono et al., 2013).

### 6.4 Antiinflammatory activity

Choi et al. used carrageenan-induced rat paw oedema model to study the antiinflammatory effect of methanol extracts from *C*. *trichotomum* leaves and found that the antiinflammatory activity of 60% methanol extract was higher than indomethacin, and that this extract also inhibited the production of PGE_2_ in RAW 264.7 cells (Choi et al., 2004). Park et al. found that methanol extract of *C*. *trichotomum* leaves inhibited the production and expression of tumor necrosis factor *α* (TNF-*α*) in mononuclear macrophages in lipopolysaccharide (LPS)-stimulated RAW 264.7 cells in a dose-dependent manner by enzyme-linked immunosorbent assay (ELISA) and reverse transcription polymerase chain reaction (RT-PCR) (Park and Kim, 2007). Electrophoretic mobility shift assay (EMSA) showed that the activity of NF-κB was also inhibited, which means that *C*. *trichotomum* inhibits the expression of the pro-inflammation gene through the inhibition of NF-κB dependent pathway in RAW 264.7 cells.

Hu et al. discovered a range of diterpenoids from the roots of *C. trichotomum* and assessed their abilities to inhibit NO production in LPS-stimulated RAW 264.7 cells. Villosin C (**15**) 15,16-dehydroteuvincenone G (**23**), 2*α*-hydrocaryopincaolide F (**35**) and trichotomin A (**46**) exerted inhibitory effects at noncytotoxic concentrations with IC_50_ values of 15.5, 6.0, 6.5 and 10.6 μM, respectively, better than the positive control. Compounds 15α-hydroxyteuvincenone E (**37**) and trichotomin B (**38**) showed moderate or weak activities with IC_50_ values of 28.9–38.6 μM (Hu et al., 2018).

Lee et al. reported that acteoside (**102**) inhibited histamine release in RBL-2H3 mast cells stimulated by melittin, arachidonic acid and toxocarotene regardless of the presence of extracellular Ca^2+^ (Lee et al., 2006). The anti-asthmatic effect on the aerosolized ovalbumin (OA) challenge in the OA-sensitized guinea-pigs of acteoside (**102**) was also evaluated (Lee J. Y. et al., 2011). **102** inhibited specific airway resistance in immediate asthmatic response, inhibited protein content at a dose of 25 mg/kg, and histamine content and PLA_2_ activity at a dose of 50 mg/kg, in bronchoalveolar lavage fluid (BALF). Kim et al. isolated three phenylpropanoid compounds from the leaves of *C*. *trichotomum,* the 80% methanol fraction and acteoside (**102**) were found to have *in vitro* and *in vivo* anti-inflammatory activity (Kim et al., 2009).

Min et al. found that apigenin-7-*O*-*β*-D-glucuronopyranoside (**84**) was more effective than omeprazole on reflux esophagitis and gastritis in mice (Min et al., 2005). Yang et al. isolated four 24-ethylcholestane derivatives from the roots of *C. trichotomum*; *in vitro* screening for anti-inflammatory activity showed that 24-ethyl-7-oxocholesta-5,22(*E*),25-trien-3*β*-ol (**91**) could dose-dependently inhibit IL-8 production in colon cancer HT-29 cells induced by IL-1*β* (Yang et al., 2014).

### 6.5 Antibacterial activity

Choi et al. tested the antibacterial activity of the MeOH extract from *C*. *trichotomum*. The n-hexane and methylene chloride (MC) fractions showed antibacterial activity against *Helicobacter pylori* at a concentration of 1.7 mg/mL and showed inhibition zones of 10 and 11 mm in disc assay, respectively. *β*-Amyrin (**70**) and 22-dehydroclerosterol (**93**), isolated from the MC fraction, revealed moderate antibacterial effects against *Staphylococcus aureus*, *Escherichia coli* and *H*. *pylori*. **70** showed clear zones of 12 and 13 mm against *E. coli* and *H. pylori*, respectively (Choi et al., 2012).

Gao et al. isolated two polyketides clerodendruketone A (**134**) and clerodendruketone B (**135**) from the leaves and twigs of *C*. *trichotomum*. The antibacterial activity evaluated by turbidimetry assay demonstrated significant antimicrobial activity of **134** (50 µg/mL) with the inhibition rate between 30%∼60% against *E. coli* and 60%∼80% against *S. aureus*, while the rates of **135** (50 µg/mL) were both between 30% and 60% (Gao et al., 2022).

### 6.6 Analgesic effect

Electric shock rat tail method (Wang and Shen, 1957) showed that the analgesic effect was shown when the injection of Chou Wu Tong (leaves and twigs of *C. trichotomum*) decoction into the abdominal cavity of mice was above 1.65 g/kg, and the peak value appeared 20–40 min after administration, and then gradually decreased, which could be maintained for 2 h. The effect before flowering was better than after flowering. The analgesic effect in mice was observed by hot plate and found that clerodendronin B, an acid soluble granular crystal of Chou Wu Tong, had a significant analgesic effect, and the effect was stronger and longer than that of morphine at a dose of 10–20 mg/Kg when injected into mice peritoneally at a dose of 400–800 mg/Kg (Xu et al., 1960b).

### 6.7 Sedative effect

Oral administration or intraperitoneal injection of the Chou Wu Tong decoction was found to have a mild sedative effect on mice, and increased doses did not induce sleep (Shen and Wang, 1957). Clerodendronin A, a water-soluble acicular crystal of Chou Wu Tong, has a strong sedative effect and has a synergistic effect with pentobarbital sodium (Xu et al., 1960b).

### 6.8 Anti HIV-1 activity

Kim et al. isolated seven phenylethanoid glycosides from the stems of *C. trichotomum*. Acteoside (**102**) and isoacteoside (**108**) showed inhibitory activities against HIV-1 integrase with IC_50_ values of 7.8 and 13.7 μM, respectively (Kim et al., 2001).

### 6.9 Others

Acteoside (**102**) and isoacteoside (**108**), isolated from *C*. *trichotomum,* showed whitening activity by the inhibition of tyrosinase activity and tyrosinase expression (Yoon et al., 2009). Clerodane-type diterpenes clerodendrin B (**48**), clerodendrin D (**49**), clerodendrin H (**53**) and clerodendrin I (**54**), were found to have insect feeding stimulant activity of the turnip sawflies, *Athalia rosae ruficornis* (Nishida et al., 1989; Nishida and Fukami, 1990; Kawai et al., 1998; Kawai et al., 1999). In addition, the sodium phosphate buffer extract of *C*. *trichotomum* leaves inhibited the growth of *Eichhornia crassipes*, causing the leaves dry, rot or even decline, which can be used for biological control of *E. crassipes* (Zheng and Lu, 2012).

## 7 Concluding remarks

*C*. *trichotomum* is endemic to East Asia and has been studied mainly in Japan, Korea and China. At present, the research of chemical composition mainly focus on terpenoids, phenylpropanoids, flavonoids and steroids. Most of the terpenoids are diterpenoids, and the main structural types are abietane-type and clerodane-type. Phenylpropanoids exist mainly in the form of glycosides. The quantity and category of volatile oil components in the literature reports are different, which may be related to different origin and extraction methods. The extracts and isolated compounds of *C*. *trichotomum* have showed many pharmacological effects such as antihypertensive, antitumor, antioxidant and anti-inflammatory, but their toxicological studies have rarely been reported.

It has been a matter of debate whether *C*. *trichotomum* belongs to the family Verbenaceae or the family Lamiaceae (Briquet, 1895; Latta, 2008). This review briefly summarises the secondary metabolites isolated from *C. trichotomum* reported in the literature. Among the 164 compounds, the largest number was abietane diterpenoids, with 39. Abietane diterpenoids are abundant in *C. trichotomum*, both in the aerial parts and the roots. From a chemotaxonomical point of view, the abundant presence of abietane diterpenoids supports the view that *C. trichotomum* transfers Verbenaceae to Labiaceae.

Studies on the chemical components of the plant mainly focus on leaves, stems, roots and fruits. So far, only one paper (Lee et al., 2016) has reported the isolation of four phenylpropanoid glycosides and a monoterpene glycoside from the flowers. However, Xu et al. found that the antihypertensive, analgesic and other activities of *C*. *trichotomum* were better before flowering than after flowering (Wang and Shen, 1957; Xu and Xing, 1962), and the reason is still unclear. Therefore, it is a very interesting and meaningful work to study the chemical compositions and pharmacological activities of flower parts and the difference between pre-flowering and post-flowering. In addition, early studies of *C*. *trichotomum* were focused on the isolation and identification of individual or several compounds from the extracts, or the pharmacological activity of crude extracts. Future research on chemical and pharmacological activities should be further closely combined to clarify the bioactive compounds of *C*. *trichotomum*. On this basis, one or several active compounds should be determined and the quality control standard of *C. trichotomum* will be established.

The active compounds isolated from *C. trichotomum* mainly included abietane diterpenoids, phenylpropanoid glycosides, flavonoid glycosides, clerodane diterpenoids and steroidal compounds, which showed excellent activities of reducing blood pressure, antitumor, antioxidant, antiinflammatory, anti-HIV-1 and promoting insect feeding. Among these ingredients, the plant’s major phytochemical is acteoside (**102**), which has rich pharmacological activities, such as anti-hypertension, antitumor, antioxidant, antiviral and whitening. Relevant studies have shown that it exists in various stages of clinical trials for anti-nephritic, hepatoprotective, and osteoarthritic activity (Srivastava and Shanker, 2022). Some of the isolates are structurally similar to the more highly active phytocompounds. However, they have not been tested for their potential biological activities. The study of the activity of analogues as well as other types of secondary metabolites is an important area for further research, as infectious diseases and civilizational diseases such as cancer require the search for new therapeutic active structures. At the same time, the active substances responsible for analgesic (Wang and Shen, 1957) and sedative (Shen and Wang, 1957) effects of *C. trichotomum* are still unclear, and Clerodendronin A (Xu et al., 1960a) and Clerodendronin B (Xu et al., 1960b), as reported crystals, need to be further purified and identified. Additionly, SAR studies were in focus of only 2 literatures (Nagao et al., 2001; Wang et al., 2013a) in the past. Therefore, the study of SAR is one of the future research directions. Of course, the chemical modifications of the isolated compounds are necessary in order to obtain semi-synthetic analogues with enhanced biological activity and improved bioavailability or safety. Recent studies have shown that there are abundant secondary metabolites associated with diverse biological activities in *C. trichotomum*. However, in numerous studies, the excellent pharmacological activity of *C. trichotomum* was established through cell culture and *in vitro* experiments. Still, an effective study through *in vivo* experiments is missing in the scientific literature. Future studies should employ appropriate animal models to elucidate the mechanism of action.

*C*. *trichotomum* is a kind of medicinal and edible plant which integrates ecological afforestation, garden greening, herbal medicine and flavor wild vegetable. But the practical application of *C*. *trichotomum* is still limited, and its pharmacological potential is also underutilized. In this paper, a comprehensive review of the literature was carried out, the information about extraction, isolation and pharmacological experiments were listed, and the isolated compounds were critically collated. The summary of the chemical compositions of *C*. *trichotomum* supports its attribution in plant classification. Compiled information on phytochemicals and pharmacological activities, as well as highlighted gaps and suggested precise directions, may contribute to the development of *C*. *trichotomum* as a drug for the treatment of disease, a Chinese herbal preparation, a plant pesticide or a functional food.
